# Transposon Mutagenesis in Chlamydia trachomatis Identifies CT339 as a ComEC Homolog Important for DNA Uptake and Lateral Gene Transfer

**DOI:** 10.1128/mBio.01343-19

**Published:** 2019-08-06

**Authors:** Scott D. LaBrie, Zoë E. Dimond, Kelly S. Harrison, Srishti Baid, Jason Wickstrum, Robert J. Suchland, P. Scott Hefty

**Affiliations:** a Department of Molecular Biosciences, University of Kansas, Lawrence, Kansas, USA; b Department of Medicine, University of Washington—Seattle, Seattle, Washington, USA; University of California, San Francisco

**Keywords:** *Chlamydia trachomatis*, genetic competence, mutagenesis, transposons

## Abstract

Transposon mutagenesis is a widely applied and powerful genetic tool for the discovery of genes associated with selected phenotypes. Chlamydia trachomatis is a clinically significant, obligate intracellular bacterium for which many conventional genetic tools and capabilities have been developed only recently. This report describes the successful development and application of a *Himar* transposon mutagenesis system for generating single-insertion mutant clones of C. trachomatis. This system was used to generate a pool of 105 transposon mutant clones that included insertions in genes encoding flavin adenine dinucleotide (FAD)-dependent monooxygenase (C. trachomatis *148* [*ct148*]), deubiquitinase (*ct868*), and competence-associated (*ct339*) proteins. A subset of Tn mutant clones was evaluated for growth differences under cell culture conditions, revealing that most phenocopied the parental strain; however, some strains displayed subtle and yet significant differences in infectious progeny production and inclusion sizes. Bacterial burden studies in mice also supported the idea that a FAD-dependent monooxygenase (*ct148*) and a deubiquitinase (*ct868*) were important for these infections. The *ct339* gene encodes a hypothetical protein with limited sequence similarity to the DNA-uptake protein ComEC. A transposon insertion in *ct339* rendered the mutant incapable of DNA acquisition during recombination experiments. This observation, along with *in situ* structural analysis, supports the idea that this protein is playing a role in the fundamental process of lateral gene transfer similar to that of ComEC. In all, the development of the *Himar* transposon system for *Chlamydia* provides an effective genetic tool for further discovery of genes that are important for basic biology and pathogenesis aspects.

## Abstract (precis)

Chlamydia trachomatis infections have an immense impact on public health; however, understanding the basic biology and pathogenesis of this organism has been stalled by the limited repertoire of genetic tools. This report describes the successful adaptation of an important tool that has been lacking in *Chlamydia* studies: transposon mutagenesis. This advance enabled the generation of 105 insertional mutants, demonstrating that numerous gene products are not essential for *in vitro* growth. Mammalian infections using these mutants revealed that several gene products are important for infections *in vivo*. Moreover, this tool enabled the investigation and discovery of a gene critical for lateral gene transfer; a process fundamental to the evolution of bacteria and likely for *Chlamydia* as well. The development of transposon mutagenesis for *Chlamydia* has broad impact for the field and for the discovery of genes associated with selected phenotypes, providing an additional avenue for the discovery of molecular mechanisms used for pathogenesis and for a more thorough understanding of this important pathogen.

## INTRODUCTION

Transposon (Tn) mutagenesis is among the more effective strategies for discovering specific genetic components associated with a given phenotype. This genetic tool has been successfully applied for a better understanding of many basic biological processes as well as for the discovery of gene products associated with host infection and pathogenesis in diverse bacterial species (1, 2). Over a decade of optimization of naturally occurring transposon systems such as Sleeping Beauty and Tc1/*mariner* has resulted in a repertoire of successful genetic insertion systems.

One of the more widely applied transposons is the hyperactive form of the *Himar1 mariner* system (3). This variant has resulted in robust transposition efficiencies and has revolutionized the study of genotype-phenotype correlation (4). The *Himar* system has many benefits that have allowed applications in phylogenetically diverse bacteria (5–11), most importantly, the simplified “cut-and-paste” mechanism that requires a single transposase for recognition of inverted repeat sequences on flanking ends of DNA. This allows direct transposition of DNA to DNA, without the requirement of additional cofactors. Additionally, the *Himar* hyperactive transposase has minimal target DNA specificity, inserting between T/A nucleobases (7, 12) and allowing relatively nonspecific insertion across an entire genome (13–15). These advantages have proven beneficial for the study of pathogenesis in a diverse set of bacteria, including obligate intracellular organisms such as *Coxiella* and *Ehrlichia*. Research in *Coxiella* has demonstrated the use of *Himar1* Tn mutagenesis to identify genes critical for cellular growth and division *in vitro* (9, 10), whereas Tn studies in *Ehrlichia* revealed genes important for mammalian infection (8). Thus far, a transposon mutagenesis system has not been comprehensively described for the globally prevalent and obligate *Chlamydia* intracellular pathogens.

Chlamydia trachomatis is the most commonly reported cause of sexually transmitted bacterial infection in the United States and worldwide. C. trachomatis infections result in a range of health issues that include pelvic inflammatory disease, sterility, blindness, and pneumonia (16, 17). C. trachomatis contains a single, circular chromosome of ∼1.04 Mb and a plasmid of ∼7,500 bp (18, 19). Lateral gene transfer has likely played a critical factor in shaping chlamydial genomes. Moreover, lateral gene transfer occurs readily between strains, and genetic exchange between C. trachomatis genomes allows the interchange of polymorphic loci, such as the immunodominant major outer membrane porin gene (*ompA*), often resulting in enhanced tissue tropism and fitness against host defenses (20–26). Despite this key role, the components that participate in and the process involved in lateral gene transfer and DNA uptake are virtually unknown in *Chlamydia.* Similarly, many aspects of basic C. trachomatis biology and pathogenesis are poorly understood. This has been largely due to the limitations of the genetic tools that have been available for *Chlamydia*.

There has been a recent surge of introductions of molecular tools and methods developed for genetic manipulation in *Chlamydia* that has been enabled by the discovery of a transformation method and effective selectable antibiotic markers (27–32). TargetTron (29) and allele-specific recombination (33, 34) are two reverse genetic tools that have been developed for targeted gene disruption and have enormous potential for functional and phenotypic studies for candidate genes. However, only chemical mutagenesis has been developed for random and unbiased strategies (35–37). This method has been effectively employed to introduce multiple-base mutations, including nonsense mutations, allowing an association of gene products with noteworthy alterations to physiological pathways (36). While chemical mutagenesis is effective, it has certain limitations, including those represented by the acquisition of multiple mutations and the challenge of correlating a phenotype to a specific genetic disruption. Furthermore, identifying the mutations requires whole-genome sequencing (WGS) and revertant or compensatory mutations may be acquired during continued passaging. Thus, the development of a random single-insertion system, such as a *Himar1* transposon containing a selectable marker, allows straightforward generation of stable, single-site mutations and simplified identification of insertion sites.

Here, the development and application of a single-plasmid *Himar1* Tn system for C. trachomatis are described. An initial subset of Tn insertion mutant strains was evaluated for potentially contrasting *in vitro* (tissue culture) and *in vivo* (mouse genital tract infection model) growth features. One of the transposon insertions occurred in a gene (*ct339*) that exhibits sequence similarity to that encoding an inner membrane DNA uptake protein termed ComEC. This mutant was investigated for the ability to perform lateral gene transfer and transformation-based DNA uptake.

## RESULTS

### Generation and genomic characterization of Tn insertion mutant clones using pCMA.

A plasmid termed pCMA (plasmid *Chlamydia* Mariner) (see Fig. S1 in the supplemental material) was designed to encode the widely utilized C9 *Himar1* transposase (3). To reduce acquisition of deleterious mutations associated with plasmid propagation in Eschericha coli, the chlamydial *ct559* promoter and ribosomal binding site were cloned upstream of the C9 transposase. The *ct559* promoter has been characterized previously (38) and exhibited transcriptional activity in *Chlamydia* but limited activity in E. coli. The *bla* gene was cloned with flanking inverted repeats for recognition and transposition by the *Himar1* transposase. Finally, the plasmid contains only the *colE1* origin of replication for plasmid propagation in E. coli, which is nonfunctional in *Chlamydia*, resulting in a nonreplicative (suicide) vector upon transformation into *Chlamydia*.

10.1128/mBio.01343-19.1 FIG S1 Plasmid map of pCMA. The C9 hyperactive variant of the *Himar1* transposon was cloned downstream of the *Chlamydia ct559* promoter. β-Lactamase (*bla*) and the corresponding promoter were cloned from chlamydial shuttle vector pGFP::SW2 (29). This antibiotic marker is flanked by *Himar1* inverted repeats to generate the transposon. Download FIG S1, PDF file, 0.1 MB. Copyright © 2019 LaBrie et al. 2019 LaBrie et al. This content is distributed under the terms of the Creative Commons Attribution 4.0 International license.

The pCMA plasmid was used in a C. trachomatis transformation procedure with β-lactams for selection (39). This procedure typically requires three passages (i.e., infection-growth-lysis-reinfection) under conditions of antibiotic selection to ensure that the resulting organisms encoding β-lactamase (*bla*) are indeed able to form infectious progeny in the presence of β-lactams. In order to decrease the potential for normally growing mutants to outcompete fitness-compromised mutant clones in a mixed infection, as well as to evaluate transformation and transposon efficiency, transformation reactions (DNA and elementary bodies [EBs]) were mixed and incubated before being split into individual wells of a 12-well plate. Each well was passaged twice with selection, and after the second passage, cultures were allowed to continue growing, with daily monitoring of C. trachomatis growth. Within 2 days of cultivation, C. trachomatis growth was observed in approximately 4 of the 12 wells, although certain samples took longer than 2 days. A total of 23 transformations were performed with 105 resistant growth cultures and a range of 0 to 9 wells with resistant growth for each transformation. These observations suggest that the transformation efficiency in C. trachomatis is very low and/or that the genome may be less tolerant of transposon insertions. This efficiency was mirrored in five independent transformations performed with pGFP::SW2 which resulted in an average of 4.8 wells (range, 4 to 8) with resistant growth (data not shown), supporting the idea that transformation efficiency likely represents the limitation rather than transposon expression or function.

To identify the site(s) of transposon insertion and other potential mutations, whole-genome sequencing was applied to the 105 mutant strains as well as to the parental L2 strain (LGV 434/Bu Hefty; CP019386.1). All mutant strains contained a single transposon insertion in an expected insertion site (T/A). The absence of mixed Tn insertion strains supports the idea that clonal isolates were obtained following the distribution of transformation sample into individual 12 wells and multiple passaging with selection.

**FIG 1:**
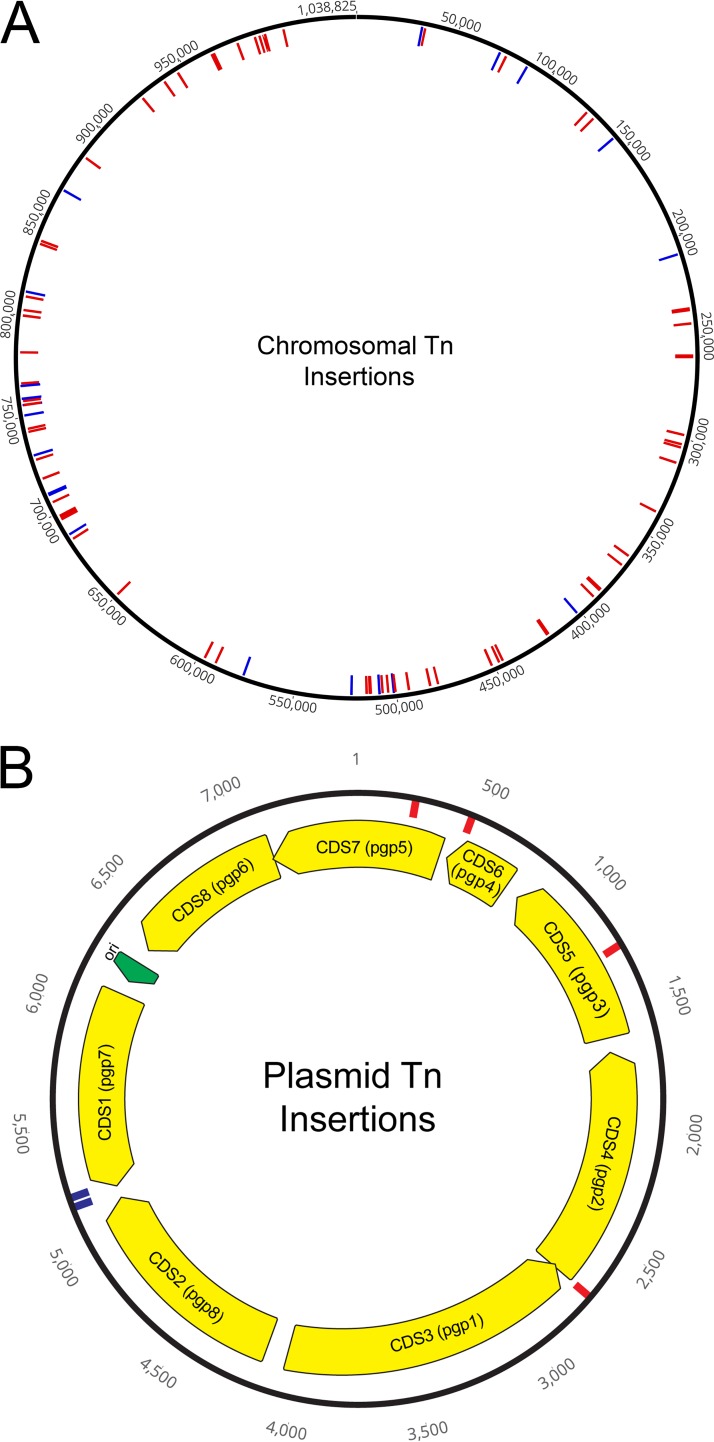
Map of transposon insertions in C. trachomatis chromosome and plasmid. (A) Sites of transposon insertions within coding regions (red) and noncoding regions (blue) throughout the C. trachomatis L2 434/Bu chromosome. (B) Sites of transposon insertions within the coding regions (red) and noncoding regions (blue) throughout the C. trachomatis L2 434/Bu plasmid. ori, origin of replication.

In total, 81 unique Tn insertion mutants were generated within coding regions (Fig. 1) (Table 1), with seven genes (*ct054*, *ct153*, *ct170*, *ct333*, *ct392*, *ct414*, and *ct550*) incurring transposon insertions in multiple sites of the coding region. While all of the studies were performed using C. trachomatis LGV 434/Bu, C. trachomatis serovar D gene nomenclature is utilized due to recognition and field familiarity. The gene *ct333* (*uvrA*) had 5 Tn insertions throughout the relatively large coding region (5,361 bp). Predominantly, Tn insertions resulted in the truncation of a protein-coding region with the addition of a short protein extension provided by the Tn insertion, depending on the insertion site and reading frame. Most of these insertions were found to have occurred within the first 60% of the coding region, providing higher confidence that the Tn insertion is disruptive to the normal protein function. Four of the insertions (*ct015*::Tn, *ct054*::Tn, *ct088*::Tn, and *ct107*::Tn; Table 1) extended the normal protein sequence, which may have little effect on protein function. Sixteen of the Tn insertions occurred in hypothetical proteins, while the rest occurred in a variety of genes with contributions to diverse biological functions. For example, five Tn insertions (*ct298*, *ct333*, *ct470*, *ct586*, and *ct575*) were involved in DNA repair mechanisms, four (*ct054*, *ct248*, *ct489*, and *ct592*) were were involved in central metabolism, and four (*ct404*, *ct540*, *ct829*, and *ct830*) encoded methyl modification enzymes. Two polymorphic membrane protein-coding regions (*ct871* [*pmpG*] and *ct414* [*pmpC*]) incurred insertions, as did both deubiquitinases (*ct867* [*cdu2*] and *ct868* [*cdu1*]). Of note, 16 of the Tn insertions occurred in genes that also incurred nonsense mutations following chemical mutagenesis (36), for a total of 54 new gene disruptions generated.

**TABLE 1:** C. trachomatis coding region transposon insertion mutants

*ctl* gene (*ct*^*c*^)::Tn *bla*	Gene name	Predicted function	Genomic position of TN insertion^*a*^	Gene insert site/total gene length (bp)	Modified protein sequence^*d*^	Sequence modification [truncated/normal length] (bp)^*d*^
*ctl0027* (*ct658* )	*sfhB*	Pseudouridine synthase	33827	544/1,008	QVKKL**X**	[Y182X; 182/335]
*ctl0063* (*ct694* )		Hypothetical	76200	17/969	SIRPTN**R**LDDKSPVX	[G8R; 16/322]
*ctl0065* (*ct696* )^*e*^		Hypothetical	79522	1,039/1,179	MRVLL**X**	[M347X; 346/392]
*ctl0101* (*ct732* )^*e*^	*ribH* (*ribE* )	6,7-Dimethyl-8-ribityllumazine synthase	124470	44/474	KDVRV**T**GWMISPRSDAQWNENSRX	[A16T; 34/157)
*ctl0105* (*ct736* )		Hypothetical phosphatidyl ethanolamine-binding proteins	129068	191/453	WIHWI**T**GWMISPRSDAQWNENSRX	[V65T; 83/150]
*ctl0125*	*murF*	UDP-N-acetylmuramoyl-tripeptide- d -alanyl- d -alanine ligase	169474	1,328/1,353	ELETL**T**GWMISPRSDAQWNENSRX	[L404T; 462/450]
*ctl0175* (*ct806* )^*h*^	*ptr*	Periplasmic Zn-dependent peptidase	223999	1,236/2,871	INSLE**Y**NRLDDKSPVX	[S422X; 422/957]
*ctl0184* (*ct813* )^*h*^		Inclusion membrane protein	235508	355/795	QFRIV**T**GWMISPRSLVAQR-35AA-YFX^*f*^	[M120T; 171/264]
*ctl0185* (*ct814* )^*e*^		Hypothetical membrane-associated protein	236256	74/300	FGGFL**T**GWMISPRSDAQWNENSRX	[A26T; 44/99]
*ctl0191* (*ct819* )		*yccA* -BAX inhibitor	242990	121/717	TSLGL**X**	[Y41X; 41/238]
*ctl0201* (*ctr829* )	*trmB*	tRNA [guanine-N(7)-]-methyltransferase	258702	68/675	IANHV**T**GWMISPRSDAQWNENSRX	[F24T; 42/224]
*ctl0202* (*ct830* )		Putative SAM-dependent methyltransferase	259440	17/585	MLTGI**T**GWMISPRSDAQWNENSRX	[D7T; 16/194]
*ctl0238b*		Hypothetical	298245	184/306	PYQYD**X**	[Y62X; 62/101]
*ctl0246* (*ct867* )	*cdu2*	Deubiquitinase and deneddylase	302887	877/1,020	QFLAW**X**	[Y293X; 293/339]
*ctl0247* (*ct868* )	*cdu1*	Deubiquitinase	304303	889/1,206	DSLYN**X**	[N297X; 297/401]
*ctl0250* (*ct871* )	*pmpG*	Polymorphic outer membrane protein	312316	786/3,039	HPTCY**F**NRRSSSKFFQKYCGRVX	[G738F; 755/1,012]
*ctl0270* (*ct015* )		Phosphate-responsive ATPase, PhoH-like protein	338386	1,277/1,305	ERSEL**T**GWMISPRSDAQWNENSRX	[A427T; 445/434]
*ctl0291* (*ct036* )^*h*^		Hypothetical, putative Inc	363864	620/1,158	LQQHL**T**GWMISPRSDAQWNENSRX	[D208T; 226/385]
*ctl0298* (*ct042* )^*h*^		Putative glycosyl hydrolase	369458	681/2,001	PSRRY**N**RLDDKSPVX	[T228N; 237/666]
*ctl0310* (*ct054* )^*h*^	*sucA*	2-Oxoglutarate dehydrogenase E1 component	385356	2,122/2,712	ARIER**X**	[Y708X; 708/903]
*ctl0310* (*ct054* )^*h*^	*sucA*	2-Oxoglutarate dehydrogenase E1 component	385932	2,699/2,712	TLFSI**T**GWMISPRSDAQWNENSRX	[G901T; 919/903]
*ctl0314* (*ct058* )		Putative membrane-spanning protein	390466	401/1,092	GWNCI**T**GWMISPRSDAQWNENSRX	[E135T; 153/363]
*ctl0339* (*ct084* )		Phopholipase D superfamily	419045	827/1,086	TPYQL**T**GWMISPRSDAQWNENSRX	[H277T; 295/361]
*ctl0343* (*ct088* )	*ssc1*	Type III secretion chaperone	423934	413/441	ELPDL**T**GWMISPRSDAQWNENSRX	[H139T; 157/146]
*ctl0361* (*ct106* )^*h*^		tRNA pseudouridine synthase A	445244	626/825	HQIRL**T**GWMISPRSDAQWNENSRX	[H210T; 228/274]
*ctl0362* (*ct107* )		A/G-specific adenine glycosylase	447004	1,089/1,107	LEKDG**N**RLDDKSPVX	[K364N; 373/368]
*ctl0367* (*ct112* )	*pepF*	Oligoendopeptidase F	450974	1,767/1,825	TSSAP**N**RLDDKSPVX	[M590N; 599/608]
*ctl0394* (*ct139* )^*h*^	*oppA*	Oligopeptide transport system binding protein	479190	336/1,281	AWEHT**N**RLDDKSPVX	[K113N; 122/427]
*ctl0398* (*ct143* )		Hypothetical	482984	708/843	TSLYS**N**RLDDKSPVX	[I237N; 246/280]
*ctl0401* (*ct146* )	*ligA*	NAD-dependent DNA ligase	487189	1,164/1,992	GINLA**N**RLDDKSPVX	[K389N; 398/663]
*ctl0403* (*ct148* )	*mphA*	FAD-dependent monooxygenase	493563	487/1,524	TPKWI**X**	[I163X; 163/507]
*ctl0408* (*ct153* )	*macP*	Membrane attack complex	499954	611/2,433	PPGTK**L**TGWMISPRSDAQWNENSRX	[T223X; 223/811]
*ctl0408* (*ct153* )	*macP*	Membrane attack complex	500482	84/2,433	SIDSD**N**RLDDKSPVX	[R29N; 38/811]
*ctl0413* (*ct157* )		Phosphatidylcholine-hydrolyzing phospholipase D	504003	956/1,206	KESAH**N**RLDDKSPVX	[T320N; 329/401]
*ctl0417a* (*ct159* )		Hypothetical	506473	118/282	MQGAY**X**	[Y39X; 39/93]
*ctl0423* (*ct170* )	*trpB*	Tryptophan synthase, beta subunit	512551	512/1,179	VNQAL**T**GWMISPRSLVAQR-35AA-YFX^*f*^	[Q172T; 223/392]
*ctl0423* (*ct170* )	*trpB*	Tryptophan synthase, beta subunit	512763	723/1,179	FFHHF**N**RLDDKSPVX	[I242N; 251/392]
*ctl0496* (*ct244* )^*h*^		Hypothetical	591198	596/1,197	VPVLI**T**GWMISPRSDAQWNENSRX	[A200T; 218/398]
*ctl0500* (*ct248* )	*glgP*	Alpha-1,4 glucan phosphorylase	596937	845/2,445	EGQEL**T**GWMISPRSDAQWNENSRX	[R283T; 301/814]
*ctl0550* (*ct298* )^*h*^	*radA*	DNA repair protein	650265	429/1,365	ISSNI**T**GWMISPRSDAQWNENSRX	[I144T; 162/454]
*ctl0578* (*ct326* )		Hypothetical	685322	670/1,545	FFVTV**T**GWMISPRSDAQWNENSRX	[S168T; 186/515]
*ctl0587* (*ct333* )^*h*^	*uvrA*	UvrABC system protein A	696123	1,850/5,361	VSIPL**T**GWMISPRSDAQWNENSRX	[G618T; 636/1,786]
*ctl0587* (*ct333* )^*h*^	*uvrA*	UvrABC system protein A	697044	2,771/5,361	ELPYL**T**GWMISPRSDAQWNENSRX	[P925T; 943/1,786]
*ctl0587* (*ct333* )^*h*^	*uvrA*	UvrABC system protein A	697874	3,601/5,361	LNHES**X**	[Y1201X; 1,201/1,786]
*ctl0587* (*ct333* )^*h*^	*uvrA*	UvrABC system protein A	698091	3,818/5,361	ESLCL**T**GWMISPRSDAQWNENSRX	[G1274T; 1,292/1,786]
*ctl0587* (*ct333* )^*h*^	*uvrA*	UvrABC system protein A	699439	5,165/5,361	LDEIA**N**RLDDKSPVX	[T1723N; 1,732/1,786]
*ctl0593* (*ct339* )^*h*^	*comEC*	Competence DNA uptake	705937	1,466/1,530	ILVYI**T**GWMISPRSDAQWNENSRX	[G490T; 508/509]
*ctl0604* (*ct350* )		Putative lipoprotein	718288	179/1701	IQPVT**R**NSRIVFTTKLSLX	[R61T; 79/566]
*ctl0611A* (*ct357* )^*h*^		Hypothetical	728274	212/333	DFSVL**T**GWMISPRSDAQWNENSRX	[R61T; 90/110]
*ctl0625* (*ct371* )		Putative outer membrane protein	742559	454/786	KANLP**L**TGWMISPRSDAQWNENSRX	[Y152L; 171/261]
*ctl0626* (*ct372* )^*h*^	*aaxA*	Porin	743881	837/1,329	KWSNL**N**RLDDKSPVX	[T280N; 289/442]
*ctl0639* (*ct383* )		Putative inner membrane protein	755828	572/732	ERYTL**T**GWMISPRSDAQWNENSRX	[L192T; 210/243]
*ctl0641* (*ct385* )		Histidine triad motif hydrolase	757770	198/336	EAGKI**N**RLDDKSP-35AA-SCX^*g*^	[I67N; 112/111]
*ctl0648* (*ct392* )^*h*^		Hypothetical	765648	762/1,131	DGPLS**I**NRLDDKSPVX	[S300X; 300/377]
*ctl0648* (*ct392* )^*h*^		Hypothetical	766400	11/1,131	MSSI**T**GWMISPRSDAQWNENSRX	[Q5T; 23/376]
*ctl0661* (*ct404* )		N6-adenine-specific DNA methylase	781874	690/828	HTPGH**N**RLDDKSPVX	[T231N; 240/275]
*ctl0671* (*ct414* )^*h*^	*pmpC*	Polymorphic outer membrane protein	799909	1,559/5,325	TNSDI**T**GWMISPRSDAQWNENSRX	[D521T; 539/1,774]
*ctl0671* (*ct414* )^*h*^	*pmpC*	Polymorphic outer membrane protein	802592	4,241/5,326	APQKG**Y**NRLDDKSPVFGSSENLRKTALQGGFF	[Q1460X; 1,460/1,775]
*ctl0671* (*ct414* )^*h*^	*pmpC*	Polymorphic outer membrane protein	802593	4,242/5,326	PQKGY**N**RLDDKSPVX	[S1455N; 1,424/1,774]
*ctl0678* (*ct421* )		Hypothetical	804491	686/702	TSPFI**T**GWMISPRSDAQWNENSRX	[L444T; 462/450]
*ctl0707* (*ct447* )	*recJ*	Single-stranded DNA-specific exonuclease	836380	1,311/1,755	DFAAG**L**NRLDDKSPVX	[M438L; 447/584]
*ctl0708a* (*ct448* )		Bifunctional preprotein translocase subunit *secDF*	838006	4,005/4,203	LQKTL**T**GWMISPRSDAQWNENSRX	[G1337T; 1,355/1,400]
*ctl0730* (*ct470* )	*recO*	DNA repair protein	863604	492/732	ESSTI**X**	[Y165X; 165/244]
*ctl0750* (*ct489* )	*glgC*	Glucose-1-phosphate adenyltransferase	884304	469/1,326	RSIVD**X**	[Y157X; 157/441]
*ctl0802* (*ct540* )		Putative tRNA [cytidine(34)-2′-O]-methyltransferase	925001	418/456	VGIVM**X**	[Y140X; 140/151]
*ctl0812* (*ct550* )		Hypothetical	938483	42/426	FRRSI**N**RLDDKSPVX	[T15N; 24/141]
*ctl0818* (*ct555* )^*e*^		ATP-dependent helicase	946408	3,260/3,600	FIGSL**T**GWMISPRSDAQWNENSRX	[L1088T; 1,106/1,199]
*ctl0837* (*ct574* )^*e*^	*pepP*	Aminopeptidase P family protein	965163	87/1,071	KDEDI**N**RLDDKSPVX	[S30N; 39/356]
*ctl0838* (*ct575* )^*e*^^,^^*h*^	*mutL*	DNA mismatch repair endonuclease	966365	626/1,731	KQGFI**T**GWMISPRSDAQWNENSRX	[E210T; 228/576]
*ctl0849* (*ct586* )	*uvrB*	Excinuclease ABC subunit B	979090	848/2,007	EGRPV**T**GWMISPRSDAQWNENSRX	[E284T; 302/668]
*ctl0853* (*ct590* )^*h*^		Hypothetical	986413	2,164/2,865	DKSAI**X**	[I722X; 722/955]
*ctl0853* (*ct590* )^*h*^		Hypothetical	988197	380/2865	RVQDL**T**GWMISPRSDAQWNENSRX	[I128T; 145/954
*ctl0855* (*ct592* )	*sdhA*	Succinate dehydrogenase flavoprotein	990457	875/1,881	PWYFL**T**GWMISPRSDAQWNENSRX	[E293T; 311/626]
*ctl0856* (*ct593* )^*e*^	*sdhC*	Succinate dehydrogenase	992076	114/862	HLLTN**N**RLDDKSPVX	[I39N; 48/287]
*ctl0858* (*ct594* )	*tatD*	Hydrolase	992950	178/792	DWMFY**N**RLDDKSPVX	[H61N; 69/261]
*ctl0867* (*ct604* )	*groEL2*	Molecular chaperone GroEL	1002459	431/1,602	LEHTV**X**	[Y144V; 144/533
*ctl_r03* /*04*		23S rRNA	136240 OR 158284	404/2,868		
*ctl0425* (*ct172* )		Pseudogene	514407			
*CDS7* (*pgp5* )	*pgp5*	Plasmid hypothetical protein	229^*b*^	165/795	SGLGA**N**RLDDKSPVX	[S52N; 61/264]
*CDS6* (*pgp4* )	*pgp4*	Plasmid hypothetical protein	460^*b*^	270/309	ALIML**N**RLDDKSPVX	[I91N; 100/102]
*CDS5* (*pgp3* )	*pgp3*	Plasmid virulence protein	1262^*b*^	331/795	ALIML**N**RLDDKSPVX	[I91N; 100/102]
*CDS3* (*pgp1* )	*pgp1*	Plasmid helicase	2715^*b*^	1,354/1,356	IKKVW**X**	[X451X; 451/451]
*CDS4* (*pgp2* )	*pgp2*	Plasmid hypothetical protein	2715^*b*^	5/1,065	MVTGW**M**ISPRSDAQWNENSRX	[N6M; 21/354]

a Position generated using C. trachomatis LGV 434/Bu (NC_010287).

b Position generated using C. trachomatis LGV 434/Bu plasmid (NC_021051.1).

c C. trachomatis serovar D (NC_000117) gene nomenclature.

d *Himar* insertion delineated by underscore; first amino acid shift delineated by bold highlighting and shown in square brackets; stop codon delineated by X. Data were calculated as follows: (protein length after insertion)/(wild-type protein length).

e Slow-growing mutants (mutants that required six or more passages to achieve visibly infectious stocks).

f Complete C-terminal sequence, QFRIVTGWMISPRSLVAQRTFEKPPCKAVFSFSEQEITRRPKRSQEDHLVNQIKYFX.

g Complete C-terminal sequence, EAGKINRLDDKSPVFGSSENLRKTALQGGFFVFRARDYAQTKTISRRSSCX.

h Denotes coding regions that were shared as nonsense mutants as reported previously by Kokes et al. (36).

Twenty-three mutant strains had Tn insertions within an intergenic region (Table 2). Many of these insertions occurred between genes with divergent coding directions as well as between those with shared coding directions, both of which could result in disruptions to gene expression. Insertions upstream of eight (*ct690*, *ct792*, *ct793*, *ct182*, *ct328*, *ct343*, *ct387*, and *ct472*) of the diverging genes occurred within 80 nucleotides (nt) of start codons. On the basis of the determinations of transcriptional start sites located on average 50 nt upstream of start codons (40) and promoters covering at least an additional 50 nt, these Tn insertions likely disrupt transcription initiation and gene expression. For insertions between genes with shared coding directions, three (*ct343*, *ct373*, and *ct423*) have transcriptional start sites reported upstream of or near (within 40 nt) the insertion potentially disrupting gene expression. Three Tn insertions also occurred between genes with convergent coding directions. While the possibility of disruption of gene expression (effecting termination or noncoding RNAs [ncRNAs]) cannot be ruled out, it is less likely that these flanking genes are affected by these Tn insertions.

**TABLE 2:** C. trachomatis intergenic region transposon insertion mutants

Flanking CTL genes (CT^*c*^)::Tn *bla*	Flanking gene orientation	Genomic position of TN insertion^*a*^	Gene insert site/total gene length (bp)	Potential gene function disruption
*ctl0024* /*0025* (*ct655* /*656* )	Converging	32332		
*ctl0059* /*0060* (*ct690* /*691* )	Diverging	72505	50/110 nt upstream of *dppD* /*phoU*	*dppD* (oligopeptide ABC transporter ATP-binding protein) and *phoU* (phosphate transport)
*ctl0070* /*0071* (*ct701* /*702* )	Converging	87381		
*ctl0111* /*0112* (*ct742* /*743* )	Shared direction	142632	98 nt upstream of *trmA* (CTL0111)	*trmA* ; 23S rRNA (uracil-5-)-methyltransferase
*ctl0160* /*0162* (*ct792* /*793* )	Diverging	208013	57/12 nt upstream of *mutS* /hypothetical	*mutS* ; DNA mismatch repair protein
*ctl0322* /*0323* (*ct076* /*077* )	Diverging	401583	176/484 upstream of hypothetical/*troA*	
*ctl0339* /*0340* (*ct084* /*085* )	Shared direction	419884	13 nt upstream of CTL0339	*CTL0339* ; Phopholipase D
*ctl0408* /*0411* (*ct154* )	Shared direction	501211	646 nt upstream of CTL0408	
*ctl0417* /*0418* (*ct161* /*162* )	Shared direction	508108	56 nt upstream of hypothetical (CTL0417)	
*ctl0433* /*0434* (*ct181* /*182* )	Diverging	522045	287/80 nt upstream of hypothetical/*kdsB*	*kdsB* ; CMP-2-keto-3-deoxyoctulosonic acid synthetase
*ctl0482* /*0483* (*ct230* /*231* )	Shared direction	576139	134 nt upstream of *yocR* (CTL0483)^*d*^	*yocR* ; Sodium-dependent transporter
*ctl0581* /*0582* (*ct327* /*328* )	Diverging	687877	161/34 nt of *trpC* /*tpiA*	*trpC* (phosphoribosylanthranilate isomerase) and *tpiA* (triosephosphate isomerase)
*ctl0596* /*0597* (*ct342* /*343* )^*e*^	Shared direction	709636	110 nt upstream of *rpsU*^*d*^	*rpsU* ; S21 ribosomal protein
*ctl0597* /*0598* (*ct343* /*344* )	Diverging	710369	13/297 nt upstream of *tsaB* /*lon*	*tsaB* ; tRNA threonylcarbamoyladenosine modification protein
*ctl0614* /*0615* (*ct360* /*361* )	Shared direction	730550	67 nt upstream of hypothetical	
*ctl0633* /*0634* (*ct378* /*379* )	Shared direction	750823	53 nt upstream *pgi*^*d*^	
*ctl0639* /*0640* (*ct383* /*384* )	Converging	755990		
*ctl0642* /*0643* (*ct386* /*387* )	Diverging	759051	218/69 nt upstream of hypothetical proteins	
*ctl0647* /*0648* (*ct391* /*392* )	Converging	765276		
*ctl0682* /*0683* (*ct423* /*424* )	Shared direction	811934	25 nt upstream of rsbV (CTL0683)	*rsbV* ; anti-sigma factor antagonist
*ctl0731* /*0733* (*ct471* /*472* )	Diverging	864902	460/18 upstream of hypothetical proteins	
*CDS1* /*2* (*pgp7* /*8* )	Converging	5189^*b*^		
*CDS1* /*2* (*pgp7* /*8* )	Converging	5232^*b*^		

a Position generated using C. trachomatis LGV 434/Bu (NC_010287).

b Position generated using C. trachomatis LGV 434/Bu plasmid (NC_021051.1).

c C. trachomatis serovar D (NC_000117) gene nomenclature.

d Transcription start site reported (40) upstream of coding region.

e Slow-growing mutants (mutants that required six or more passages to achieve visibly infectious stocks).

Tn insertions also occurred within the chlamydial plasmid. Four (*pgp1*, *pgp2*, *pgp4*, and *pgp5*) of the coding regions incurred an insertion, although the Tn insertion in *pgp1* was at the second to last codon and may have little effect on the protein generated. Two Tn insertions were also found in the intergenic region of the convergent *pgp7* and *pgp8* genes. Two prior studies have investigated the requirement of individual genes for plasmid maintenance (41, 42). Both studies supported the idea that products of the *pgp1*, *pgp4*, and *pgp5* genes are not required; however, the presence of almost all of the *pgp2* coding region was found to be required for plasmid maintenance. As such, it was unexpected for a Tn insertion in *pgp2* which results in a severely truncated coding region to be identified. This may indicate that there is a product (e.g., ncRNA) within the coding region, instead of the expected Pgp2 protein product, that is key for plasmid replication. In support of this, the *pgp2*::Tn mutant strain was grown without antibiotics for two passages and the plasmid was found to still be present by PCR analysis (data not shown).

### *In vitro* growth characteristics of transposon mutants.

**FIG 2:**
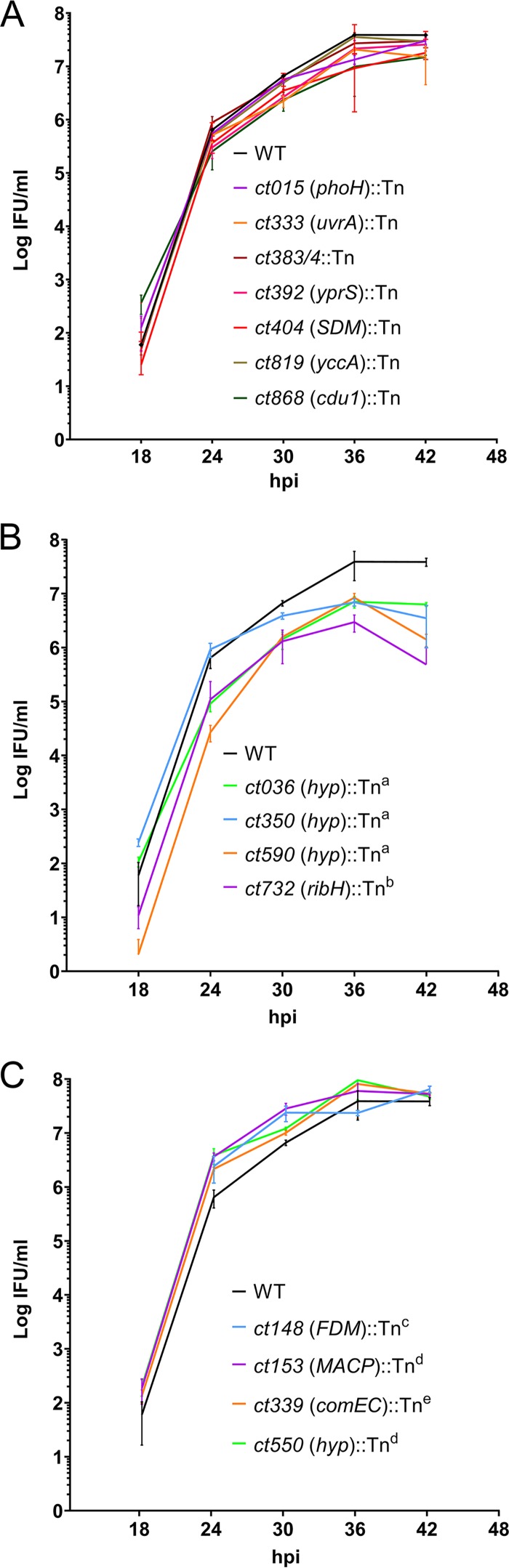
Progeny production of Tn mutants from 18 to 42 hpi. L929 cells were infected with the WT (parental) strain or a Tn insertion mutant clone (gene with Tn insertion is indicated) in triplicate. Quantities of inclusion-forming units (IFU) were determined from lysates at 18, 24, 30, 36, and 42 hpi. Mutants showed progeny production patterns similar to those of the WT parental L2 clone (A), those with significantly decreased progeny production at multiple time points (B), or those with significantly enhanced progeny production at one or more time points (C). Data are shown as means and SD of results from triplicate wells. Statistical significance was calculated using Student’s two-tailed unpaired *t* test with a *P* value of <0.05. Progeny production was significantly reduced at 24, 30, and 42 hpi (indicated with a superscript “a”) or at 24, 30, 36, and 42 hpi (indicated with a superscript “b”). Progeny production was significantly increased at 30 and 42 hpi (indicated with a superscript “c”) or 24 and 30 hpi (indicated with a superscript “d”) or 24, 30, and 42 hpi (indicated with a superscript “e”).

**FIG 3:**
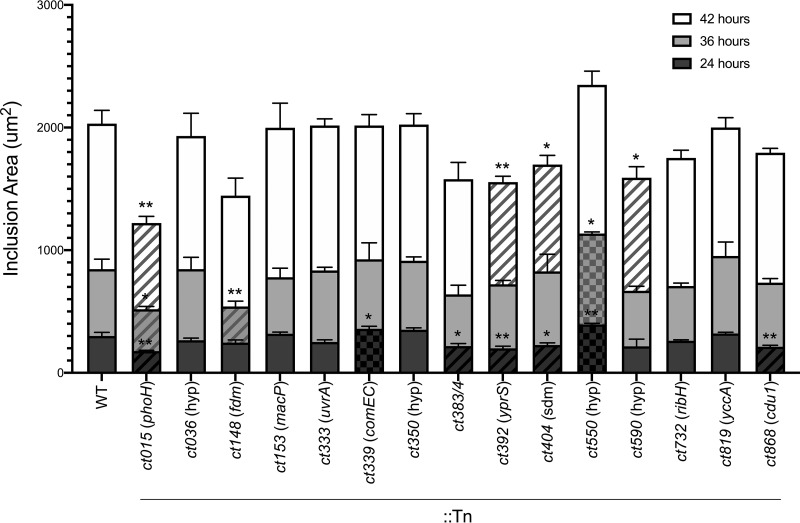
Inclusion sizes of Tn mutant strains. Inclusion size was measured at 24, 36, and 42 h postinfection for each Tn mutant and parental strain (WT). Mutant strains are displayed as stacked bars showing inclusions larger than the WT inclusions (checked), equal to the WT inclusions in size (solid), or smaller than the WT inclusions (striped). Triplicate samples were evaluated, with 250 to 1,000 inclusion areas measured per sample. **, *P* < 0.005; *, *P* < 0.05 (unpaired Student's *t* test).

While most of the transposon mutant clones did not exhibit noticeably slow growth or poor production of infectious progeny during initial selection and tissue culture propagation, more-subtle growth rates or morphologic differences may be associated with certain mutants. To discover potential defects in infection, growth, or developmental cycle processes associated with transposon mutant clones, temporal analyses of infectious progeny production and bacterial morphologies and quantitative assessment of inclusion sizes were performed for a subset of 16 Tn mutants (Fig. 2; see also Fig. 3 and Fig. S2 and S3). In addition to the parental strain, a clone with a Tn insertion between converging genes (*ct383*/*4*::Tn) was also evaluated.

10.1128/mBio.01343-19.2 FIG S2 Confocal microscopy of Tn mutants at 24, 36, and 42 hpi. L929 cells were infected with WT and mutant C. trachomatis at 24, 36, or 42 hpi. Samples were stained with DAPI (blue), and a C. trachomatis culture confirmation test was performed for major outer membrane protein (MOMP) (green) and host cytosol (red). Images were captured at ×150 magnification, with a single z plane displayed. Bar, 10 μm (consistent within time points). Download FIG S2, PDF file, 1.7 MB. Copyright © 2019 LaBrie et al. 2019 LaBrie et al. This content is distributed under the terms of the Creative Commons Attribution 4.0 International license.

10.1128/mBio.01343-19.4 FIG S4 Postinfection titers of inocula for infectious progeny assay. The titers of the inocula used for the progeny production assay were measured to reflect the specific dose used to infect the 24-well plates. L929 cells were infected with inocula in a 96-well plate and incubated at 37°C and 5% CO_2_. At 24 h postinfection, cells were washed, fixed with methanol, washed, and stained with DAPI (nucleic acid) and a Chlamydia trachomatis culture confirmation test was performed to view inclusions (anti-MOMP) and to stain the host cytosol. Titers of samples were determined to measure the levels of inclusion-forming units (IFU) per milliliter. Download FIG S4, PDF file, 0.2 MB. Copyright © 2019 LaBrie et al. 2019 LaBrie et al. This content is distributed under the terms of the Creative Commons Attribution 4.0 International license.

Analyses of the production levels of infectious progeny (EB) of parental (wild-type [WT]) and individual Tn mutant clones were performed on lysates in 6-h increments between 18 and 42 h postinfection (hpi) (Fig. 2). Progeny production levels reflect bacterial replication as well as ability to convert into the infectious EB form. Defects in either of these can be detected with this approach, enabling a more focused analysis of candidate mutant strains. Three distinct growth phenotypes were observed among Tn mutants; normal, decreased, and increased progeny production relative to the parent strain. Almost half (7 of 16) of the Tn mutants displayed EB production levels and timing similar to those seen with the WT, as did the clone with a Tn insertion between two converging genes (*ct383*/*4*::Tn) (Fig. 2A). However, four mutants (*ct036*::Tn, *ct350*::Tn, *ct590*::Tn, and *ct732*::Tn) displayed significant deficiencies in progeny production at two or more time points (Fig. 2B). At 24 hpi, strain *ct590*::Tn (hypothetical) displayed progeny production levels that were almost 2 log-fold lower whereas *ct036*::Tn and *ct732*::Tn (*ribH*) mutants displayed progeny production levels that were about 1 log-fold lower. An interesting observation was the enhanced progeny production of four mutant clones (*ct153*::Tn, *ct148*::Tn, *ct339*::Tn, and *ct550*::Tn), which was most notable at 24 hpi. Clones *ct153*::Tn and *ct148*::Tn maintained this enhanced level at 30 hpi, with all of the mutant clones matching the parental clone at 36 and 42 hpi (Fig. 2C). Of the Tn mutants evaluated, *ct696*::Tn displayed the most contrasting and slowest growth phenotype, with propagation requiring up to 5 days before passage was successful and with undiluted cell lysate and levels of inclusion-forming units (IFU) below 10/ml (data not shown). Overall, progeny production analysis revealed that defects were observed for many of the Tn insertion clones, albeit most of the data were within a log-fold difference range.

10.1128/mBio.01343-19.3 FIG S3 Confocal microscopy of *ct696*::Tn mutant. L929 mouse fibroblasts were infected with either C. trachomatis parental or *ct696*::Tn mutant cells. Infections were fixed at 24 hpi and subjected to immunofluorescence staining. Images were acquired at ×150 magnification with deconvolution for enhanced image resolution. (A) WT C. trachomatis inclusion showing normal RB division (white arrow). (B) *ct696*::Tn showing normal RB division (white arrow). (C) Dispersed localizations of *ct696*::Tn within the cytosol. (D) Aggregating form of *ct696*::Tn. Coccoid RBs (blue arrow) that are external to the aggregation (yellow arrow) are shown. Blue, DAPI (nucleus); red, Evans blue (cytoplasm); green, OmpA (C. trachomatis organisms). Bar, 1 μm. Download FIG S3, PDF file, 0.3 MB. Copyright © 2019 LaBrie et al. 2019 LaBrie et al. This content is distributed under the terms of the Creative Commons Attribution 4.0 International license.

Defects in the temporal expansion of an inclusion could suggest that a gene product plays a role in many aspects of chlamydial growth, and so, the area of inclusions was calculated at 24, 36, and 42 hpi. The data shown in Fig. 3 highlight that the inclusions for 5 Tn mutants (*ct015*::Tn, *ct383*/*4*::Tn, *ct392*::Tn, *ct404*::Tn, and *ct868*::Tn) were significantly smaller than that for the parental strain at 24 hpi. Only mutant *ct015*::Tn maintained smaller inclusion sizes at 36 and 42 hpi, with *ct392*::Tn, *ct404*::Tn, and *ct590*::Tn displaying smaller inclusions at 42 hpi. Two Tn mutants (*ct339*::Tn and *ct550*::Tn) had inclusions larger than the parental inclusions at 24 hpi, with the *ct550*::Tn inclusions maintaining a larger size than the parental inclusions at 36 hpi. Interestingly, *ct550*::Tn was the only mutant to display a correlation between progeny production and inclusion size (Fig. 2C; see also Fig. 3), both of which showed increases relative to the parental data.

To discover if any abnormal morphologic phenotypes (bacterial or inclusion) are associated with this subset of transposon mutant clones, confocal microscopy was applied to immunostained parental and transposon mutants at 24, 36, and 42 hpi (Fig. S2). Almost all of the Tn mutants displayed inclusions that were localized near the nucleus as well as general shapes of the inclusion and reticulate bodies (RBs) that were similar to those seen with the parental strain. Notable exceptions included mutant *ct048*::Tn, which contained larger but fewer RBs within the inclusion at 42 hpi. Similarly to the growth assessments, the *ct696*::Tn inclusions and bacterial morphologies were among those that were the most highly contrasting. The inclusions were ill defined, and the few reticulate bodies present were dispersed in the cytoplasm (Fig. S3).

In all, cell culture growth and phenotype assessments revealed that many Tn mutant strains displayed defects that represented significant differences from the parental strain. However, these defects were relatively subtle, with peak EB titers fluctuating by approximately with 1 log fold, and inclusion sizes were often within 10% to 15% of sizes seen with the parental strain, although the inclusions seen with the *ct015*::Tn mutant were over 30% smaller than the parental inclusions. The extreme exception to these trends was mutant *ct696*::Tn, with gross defects in EB production and in bacterial and inclusion morphologies. Moreover, there was little correlation between EB production titers and inclusion sizes, with the exception of the hypothetical *ct550* encoding gene, which displayed more and larger inclusions.

### *In vivo* assessment of infectivity.

While the majority of the Tn mutants analyzed showed minimal morphologic or growth differences under *in vitro* cell culture conditions, some gene products may be of greater significance under the more physiologically relevant, diverse, and challenging conditions associated with mammalian infection. In order to assess this, a mouse model of C. trachomatis infection was implemented. Unfortunately, C. trachomatis L2 administered vaginally in mouse infections is readily cleared, rarely and unreliably ascends to the upper genital tract, and is not associated with the development of pathology (43, 44). As such, the transcervical model was used to deliver infectious doses directly into uterine horns of the upper genital tract (45). Previous experiments have shown that transcervical C. trachomatis infection of uterine horns typically peaked between 3 and 6 days postinfection and that the bacteria cleared (nearly 3 log-fold) after ∼9 days postinfection (45). Therefore, to discover potential deficiencies in infection and replication in the upper genital tract, mice were infected with individual Tn mutants and harvested on day 5, within the period of the peak of C. trachomatis L2 infection.

**FIG 4:**
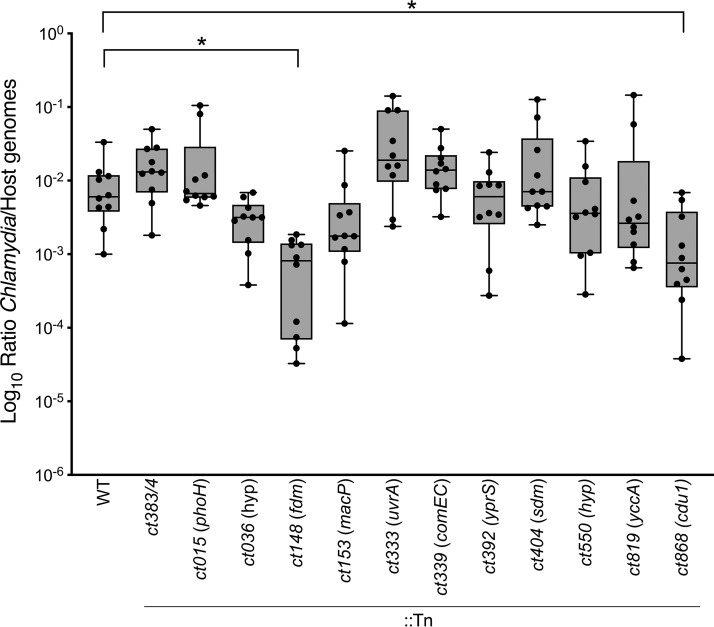
*In vivo* infections using Tn mutants with a transcervical mouse infection model. Groups of 10 female C57BL/6 mice were infected transcervically with 5 × 10^5^ IFU of either parental (WT) C. trachomatis or Tn mutants as indicated. Five days postinfection, genital tracts were harvested and DNA was purified from uterine horns. Bacterial burdens were calculated as levels of *Chlamydia* genomes relative to host genomes, and ratios are shown as box-and-whisker scatter plots representing data from 10 mice. *, *P* < 0.05 (unpaired Student's *t* test).

Twelve Tn mutants that were evaluated for growth and morphology, including the parental strain and *ct383*/*4*::Tn clones, were used to infect mice and assess bacterial burden (Fig. 4). Most of the Tn mutants displayed infection levels similar to those seen with the parental C. trachomatis strain; however, two Tn mutant clones showed statistically significant decreases in levels of detectable organisms in the uterine horns compared to parental L2 (*P* < 0.05; Fig. 4). Disruptions in a FAD-dependent monooxygenase (FDM) (*ct148*) and the *cdu1* deubiquitinase (*ct868*) were followed by a nearly 0.5 log-fold to 1 log-fold decrease in the levels of detectable organisms compared to the WT. Mutant *ct148*::Tn showed a striking deficiency with respect to detectable organisms, with 4 of 10 infected animals showing infection levels falling 2-log below the median of infection for WT-infected animals. Interestingly, mutant *ct148*::Tn displayed a slightly enhanced level of production of infectious progeny *in vitro* (Fig. 2C) relative to the parental strain. Conversely, mutant *ct868*::Tn showed no decreased growth rates *in vitro*, suggesting that *cdu1* is dispensable for optimal growth in cell culture but that the absence of the corresponding gene product is important for growth *in vivo.* Importantly, the bacterial burdens seen with *ct868*::Tn infections were very similar to the levels previously reported from a study performed with this mutant strain (46), providing additional confidence in the reproducibility of these observations. While the results were not statistically significant, two additional mutants exhibited a noticeable decrease in bacterial burden, namely, mutants *ct036*::Tn (hypothetical) and *ct153*::Tn (membrane attack complex/perforin [MACPF]). Mutant *ct036*::Tn was among the clones that displayed lower levels of progeny production whereas mutant *ct153*:Tn produced more infectious progeny than the parental clones.

Clearly, further temporal and spatial analyses in mice, detailed molecular and cellular studies, and genetic complementation are required for a more complete understanding of the role and function of gene products with demonstrated deficiencies in mice. However, these comparative *in vitro* and *in vivo* observations highlight that certain gene products may exhibit a more pronounced fitness defect in mice, prompting a focus on these gene products.

### *In silico* analyses of CT339 support functional identification of CT339 as DNA uptake protein ComEC.

*Chlamydia* bacteria have been demonstrated to acquire and integrate DNA within or between certain *Chlamydia* species (24, 25, 47–49). Given the expected importance of lateral gene transfer in the evolution and adaptation of *Chlamydia*, as well as the paucity of identifiable gene candidates that may play a role in this fundamental process, the Tn insertion in *ct339* was of particular interest for a more in-depth analysis among the members of the subset of Tn mutants. CT339 shares similarity with multiple competence-associated protein families and conserved domains, including ComEC (E values ranging from 2.33e−03 to 4.40e−24). In both Gram-negative and Gram-positive bacteria, ComEC plays a key role as an inner membrane protein, transporting single-stranded DNA (ssDNA) into the cytosol of the bacterial cell during natural DNA acquisition (50, 51). To further investigate the hypothesis that *ct339* encodes a ComEC homolog, *in silico* analyses were performed.

ComEC is an integral membrane protein that typically has 9 to 12 transmembrane helices and a conserved metal-binding motif, HΦxxΦSGΦH (“Φ” indicates hydrophobic residues; Fig. S5A) (50). Topology modeling of CT339 using five hydropathy programs resulted in a range of 8 (SPLIT) to 11 (MEMSAT) predicted transmembrane segments, which is similar to the proposed 9 to 12 transmembrane regions of ComEC (50). As previously described for ComEC (50), HMMTOP prediction identified a large N-terminal loop within CT339 spanning residues 94 to 256 with a potentially stabilizing disulfide bond formed by C148-C167, as well as the competence domain containing the conserved metal-binding motif HΦxxΦSGΦH. For all five hydropathy programs, an extracellular N-terminal domain and intracellular C-terminal domain were predicted, similar to those of ComEC (50). In contrast to the predicted topology of ComEC, none of the predictive transmembrane helices for CT339 display an amphipathic character and no segments are predicted to be buried parallel to the membrane surface (Fig. S5B) (50). On the basis of these *in silico* data, a transmembrane topology model was designed (Fig. S5C) supporting the structural homology between CT339 and ComEC, specifically within conserved competence domains and the N-terminal loop. Using this model, the Tn insertion site was identified within the last transmembrane segment, eliminating G490 through the carboxyl end of the protein, L509. It has been proposed that ComEC from *Bacillus* exists as a homodimer within the bacterial membrane and forms a pore for DNA uptake (50). Consequently, disruption of either the N-terminal loop or the C-terminal loop, as is observed with *ct339*::Tn insertion, may prevent proper folding and formation of this pore, therefore abolishing the ability to acquire DNA.

10.1128/mBio.01343-19.5 FIG S5 CT339 has a predicted metal binding motif and topology similar to ComEC. (A) Multiple-sequence alignment of 10 competence proteins identified as containing the multidomain ComEC from the Conserved Domain Database. COG0658 was used to identify 10 bacterial species with similar competence domains with an E value of <6e−3. Hashtags indicate conserved histidine metal-binding motifs. Conserved hydrophobic residues are highlighted in orange. (B and C) Hydropathy plots of (B) ComEC from Bacillus subtilis and (C) CT339 from *Chlamydia*. Both predicted competence proteins display a large extracellular N-terminal loop stabilized by two cysteines (highlighted in yellow) and an extended C-terminal loop. The competence domain identified by analysis of data from the Conserved Domain Database is highlighted in blue, and the transposon insertion site is highlighted in green. Download FIG S5, PDF file, 0.2 MB. Copyright © 2019 LaBrie et al. 2019 LaBrie et al. This content is distributed under the terms of the Creative Commons Attribution 4.0 International license.

### Requirement of CT339 for DNA uptake via lateral gene transfer.

**FIG 5:**
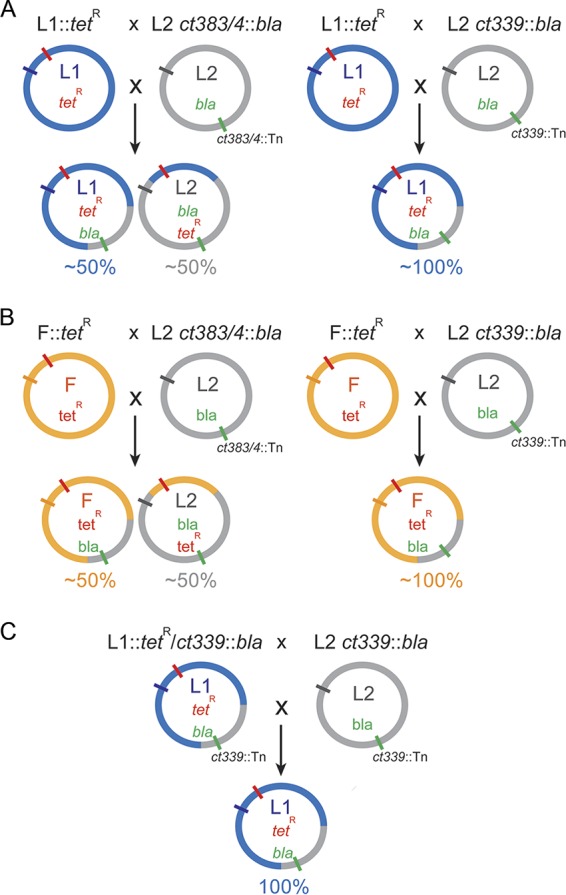
Graphic depiction of intraspecies recombination to evaluate functional role of CT339 in lateral gene transfer. (A) Parental C. trachomatis with L1 *ompA* (blue circle, blue tick) encoding *tet*^r^ (red tick) crossed with C. trachomatis with L2 *ompA* (gray circle, gray tick) containing either *ct383*/*4*::Tn *bla* (green tick) or *ct339*::Tn *bla* (green tick) to enable lateral gene transfer of resistance markers and selection of dual resistance chimeric clones (blue and gray circles). (B) Similar experiment using parental C. trachomatis with F *ompA* (yellow circle, yellow tick) encoding *tet*^r^ (red tick) crossed with C. trachomatis with L2 *ompA* (gray circle, gray tick) containing either *ct383*/*4*::Tn *bla* (green tick) or *ct339*::Tn *bla* (green tick). Serovar-specific antibodies revealed the relative distributions of resulting dually resistant chimera organisms (yellow and gray circles). Quantitative evaluations of resulting chimeras are provided in Table 3. The resulting mix of L1 (blue) and L2 (gray) or of F (yellow) and L2 (gray) reflects the almost equal amounts of resulting OmpA serovar seen when the control *ct*383/4::Tn was used. In contrast, virtually all of the resulting organisms contained a L1 (blue) or F (yellow) *ompA* when *ct339*::Tn was used, indicating that the *tet*^r^ gene, near *ompA*, was unable to be transferred into the L2 *ct339*::Tn mutant (gray). (C) Recombinants resulting from the experiment represented in panel A containing L1 *ompA* (blue circle, blue tick) encoding *tet*^r^ (red tick) and the *ct339*::Tn *bla* (green tick) were crossed with the L2 *ct339*::Tn *bla* mutants. All resulting progeny contained the L1 *ompA* (blue tick).

As previously mentioned, the transposon insertion in *ct339* provided an opportunity to experimentally evaluate the importance of this gene product in lateral gene transfer and to support the *in silico* prediction of CT339 as a functional homolog to ComEC. Lateral gene transfer, including the transfer of specific antibiotic resistance genes, has been demonstrated to occur readily during coinfections with C. trachomatis strains (intraspecies) that encode different OmpA serotypes (25, 47). To evaluate the importance of CT339 in lateral gene transfer, intraspecies coinfections with different parental OmpA serovar strains (L1 or L2) encoding different antibiotic markers (L1 *tet*^r^ or L2 *bla*) under conditions of dual antibiotic selection were performed (Fig. 5A). Each L2 strain used had either an intact (*ct383*/*4*::Tn *bla*) or a disrupted (*ct339*::Tn *bla*) *ct339* gene. The relative levels of genetic transfer, particularly of the antibiotic marker, of the OmpA serovar strains were assessed by determining the quantity of progeny generated that exhibited each parental OmpA serotype (e.g., *L1* versus *L2*).

Intraspecies coinfections and dual antibiotic selection with tetracycline-resistant C. trachomatis L1 (L1::*tetR*) and either the transposon control mutant L2 *ct383*/4::Tn *bla* or the proposed ComEC homolog disrupted clone L2 *ct339*::Tn *bla* were performed (Fig. 5) (Table 3). When C. trachomatis L1::*tet*^r^ and L2 *ct383*/4::Tn *bla* were used for coinfections, high levels (>6 × 10^5^ IFUs) and nearly equal amounts of recombinant progeny displaying either parental OmpA (58% L1 and 42% L2) were observed. The relatively equal frequencies support the idea that DNA uptake and lateral transfer of the *tetR* gene from the L1 strain into to L2 genome occurred as efficiently as that of the *bla* gene from the L2 strains into the L1 strain. In contrast, when the same L1::*tet*^r^ parent was used in a coinfection with L2 *ct339*::Tn *bla*, similar levels of L1 OmpA-expressing progeny with dual resistance were detected (5.17 × 10^5^ IFUs) but nearly 100-fold-lower levels of progeny displaying L2 OmpA were observed (7 × 10^3^ IFUs) (Table 3). This supports the idea that DNA uptake and transfer of the *bla* gene into L1, which has an intact *ct*339 gene, were as efficient as the previous cross. In contrast, *tetR* was found to be incorporated into the L2 genome with the *ct339*::Tn *bla* mutation at a 2 log-lower level. While this supports the idea of the importance of CT339 (*comEC*) in DNA acquisition, the low level of *tetR* resistance suggests that the *comEC* gene product may not be essential for the process.

**TABLE 3:** Frequency of C. trachomatis serotype (OmpA) following coinfection and dual antibiotic selection

C. trachomatis genotype	C. tractomatis serotype and frequency (%)		
L1::*tet*^r^ × L2 *ct383*/*4*::Tn *bla*	L1 OmpA; 8.67 (±0.58) × 10^5^	L2 OmpA; 6.33 (±0.58) × 10^5^	L2 OmpA progeny; 42.2 (±1.99)
L1::*tet*^r^ × L2 ct339::Tn *bla*	L1 OmpA; 5.17 (±0.43) × 10^5^	L2 OmpA; 7.00 (±1.00) × 10^3^	L2 OmpA progeny; 1.12 (±0.40)
F::*tet*^r^ × L2 *ct383*/*4*::Tn *bla*	F OmpA; 4.33 (±1.15) × 10^5^	L2 OmpA; 3.33 (±0.58) × 10^5^	L2 OmpA progeny; 44.0 (±6.26)
F::*tet*^r^ × L2 *ct339*::Tn *bla*	F OmpA; 1.33 (±0.61) × 10^4^	L2 OmpA; 5.33 × 10^0^ (±1.53)	L2 OmpA progeny; 0.05 (±0.01)

**FIG 6:**
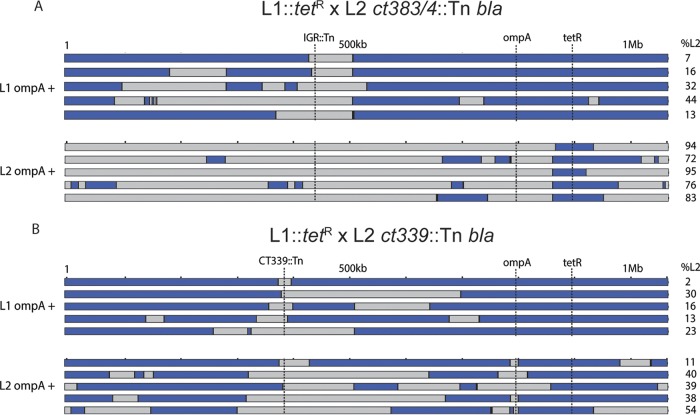
Schematic representation of regions and genome compositions of C. trachomatis recombinant clones following lateral gene transfer with selected Tn mutants. Blue bars represent the regions of the genome from the L1/*tet*^r^ parent. Gray bars represent regions from either L2 *ct383*/*4*::Tn *bla* or L2 *ct339*::Tn *bla* parent. Percentages of the C. trachomatis L2 genome present are indicated (right). (A) Progeny genomes from crosses between tetracycline-resistant C. trachomatis L1 and *ct383*/*4*::Tn *bla*. (B) Progeny genomes from crosses between tetracycline-resistant C. trachomatis L1 and *ct339*::Tn *bla*.

An alternative hypothesis to explain the low level of L2 OmpA-positive samples seen following the L1::*tetR* and L2 *ct339*::Tn *bla* crosses is that, instead of poor uptake of the *tetR* gene into L2 *ct*339::Tn *bla* organisms, a region of DNA that included the L2 *ompA* gene and the *ct*339::Tn *bla* marker was transferred and integrated into L1 parent organisms. This would “convert” the L1 to an L2 OmpA serotype. To investigate this hypothesis, whole-genome sequencing was performed on several individual recombinants from each coinfection (L1::*tet*^r^ versus L2 *ct339*::Tn *bla* or L2 *ct383*/4::Tn *bla*). As indicated in Fig. 6A, L1::*tet*^r^ and L2 *ct383*/4::Tn *bla* crosses yielded genomes that predominantly reflected the OmpA serotype (mostly blue for L1 OmpA and mostly gray for L2 OmpA). Specifically, L1 or L2 OmpA-positive samples had an average of 78% L1 or 84% L2 genomic composition, respectively. In contrast, L2 OmpA-positive samples generated after a L1::*tet*^r^ × L2 *ct339*::Tn *bla* coinfection had minimal L2 genome in the resulting clones (L2 [gray]; Fig. 6B), with an average genomic L2 composition of 36%, representing less than half the level observed in L1::*tet*^r^ × L2 *ct383*/4::Tn *bla* crosses. Matching the levels of antibiotic resistance transfer in *ct*339 intact samples (L2 *ct383*/4::Tn *bla*), L1 positive OmpA samples showed an average of 80% L1 genomic composition (blue; Fig. 6B). These data support the hypothesis that DNA regions containing an L2 *ompA* gene and the *ct339*::Tn *bla* marker were most likely acquired by L1 parent clones through lateral gene transfer instead of *tetR* acquisition of a L2 *ompA ct*339::Tn *bla* organism.

The generation of L1::*tet*^r^/*ct339*::Tn *bla* recombinant clones provided an additional opportunity to further investigate the essentiality of *ct339* for DNA acquisition and genome incorporation. These clones were used in coinfections with the parent L2 *ct339*::Tn *bla* clone with dual antibiotic selection (Fig. 5C). If *ct339* were essential for DNA acquisition and incorporation, then all resulting clones should be only L1 OmpA positive, as the L2 *ompA* gene would not have the ability to be transferred and incorporated into the L1 genome to enable a serotype conversion. However, if *ct339* is only partially required, then a mix of L1 and L2 OmpA populations would be expected to be observed following coinfection and antibiotic selection (i.e., with the *tetR* marker taken up and incorporated into the L2 genome or L2 OmpA transferred and incorporated into the L1 genome). Three independent coinfections revealed that only L1 OmpA-positive inclusions were observed with extensive (∼5 × 10^7^) levels of IFUs utilized per evaluated cross (data not shown). Additionally, transformation of the L2 *ct339*::Tn *bla* mutant was also attempted using an inducible green fluorescent protein (GFP) plasmid (pTLR2-GFP) and chloramphenicol for selection. Every attempt to transform parental L2 samples that had an intact chromosomal *ct339* gene was successful using the inducible plasmid. In contrast, none of the attempts to transform L2 *ct339*::Tn *bla* with the plasmid were successful (data not shown).

To further address the functionality of CT339 in lateral gene transfer, similar coinfections and dual-selection experiments were performed with tetracycline-resistant C. trachomatis serovar F (F::*tet*^r^). Using strains that are more phylogenetically distant (52) reduces the challenge of delineating highly similar genomic crossover regions. Three independent crosses and selections were performed with C. trachomatis F::*tetR* and either C. trachomatis L2 *ct383*/4::Tn *bla* or C. trachomatis L2 *ct*339::Tn *bla* (Fig. 6B). As observed in the L1 × L2 *ct383*/4::Tn *bla* experiments (Fig. 6A) (Table 3), approximately half of the inclusions with an intact *ct339* exhibited either OmpA F (56%) or L2 (44%) seroreactivity (Table 3). Similarly to the L1::*tet*^r^ versus L2 *ct*339::Tn crosses, more than 99.94% of the F::*tet*^r^ × L2 *ct339*::Tn inclusions were positive for only OmpA F (Table 3). Taken together, these data support the hypothesis that *ct339* is critical for DNA uptake and is likely serving as a ComEC functional homolog in C. trachomatis.

## DISCUSSION

There have been many recent firsts in the area of genetically modifying Chlamydia trachomatis, including chemical mutagenesis (35–37), group II intron gene disruption systems (29), allele replacement (33, 34, 53), inducible gene expression (28, 54), and targeted gene repression (32). The development and validation of a transposon mutagenesis system using the *Himar1* transposase, as presented here, provide another first and a key advance for the *Chlamydia* field. The data presented demonstrate that the transposase is functional within *Chlamydia* and that the use of the nonreplicative plasmid results in mutant C. trachomatis clones containing a single genomic insertion. The insertion sites are readily discovered and can be characterized by common PCR-sequencing-based techniques. These insertion mutations are stable, even without antibiotic selection, limiting the chances of genetic reversion. Support for this stability was provided by the multiple passages and large-scale growth conditions (3 to 5 passages for expansion and spinner flask cultivation), without antibiotics, employed prior to animal studies as well as by the confirmation of Tn insertions in samples prior to and after animal infections. A primary benefit of this single-insertion, random mutagenesis approach is the ability to associate a specific genetic disruption with a resulting phenotype. While polar effects can occur as the result of a Tn insertion, such effects can be viewed as a benefit, as an insertion can enable researchers to focus on a selected but known pool of candidates associated with a particular phenotype. As demonstrated by the insertion disruption of ComEC homolog CT339 and by the subsequent biological studies related to lateral gene transfer in *Chlamydia* presented here, the scientific contributions of this molecular tool and approach are expected to be very useful for *Chlamydia* studies.

Notwithstanding these benefits, the present report also highlights the relatively low efficiency associated with *Chlamydia* transposon mutagenesis and its current limitations for large-scale studies. While most of this restriction is thought to be due to the extremely poor transformation efficiency associated with C. trachomatis, the low insertional efficiency may also suggest that high numbers of genes are essential for growth and completion of the chlamydial developmental cycle under cell culture conditions. A total of 70 genes incurred a Tn insertion in this study, while the results of a random chemical mutagenesis study performed by Nguyen and Valdivia (37) indicated that 84 protein-coding genes incurred a nonsense mutation with limited *in vitro* growth effects. Fifteen of the 84 genes were also disrupted in that Tn insertion analysis, providing a total of 139 genes that have disruptions in the coding region following ethyl methanesulfonate (EMS) mutagenesis or Tn insertion. This represents around 15% of the encoding gene products. While the number of genes that are essential for growth under tissue culture conditions is currently unknown, the relative low level of overlap of disruptive Tn and EMS mutations suggests that many more C. trachomatis genes may tolerate disruption.

Among the more striking observations were the enhanced *in vitro* growth of a *ct148*::Tn mutant clone and the almost log-fold decreased infectivity in mice (Fig. 2C; see also Fig. 4). The Tn insertion occurs in the first third of the coding region, providing higher expectations that the function of this gene product is indeed disrupted. The gene downstream from *ct148* is oriented in the opposite direction, although there is a gene 200 bp upstream. This distance and the reported location of the transcriptional start site 85 bp upstream of *ct148* (40) support the idea that there is a single gene operon and that polar effects from this Tn insertion are limited. The *ct148* gene encodes a FAD-dependent monooxygenase (FDM). Monooxygenases are known to play roles in multiple metabolic pathways, and, as Iliffe-Lee and McClarty showed previously (55), C. trachomatis contains an almost complete and functional Embden-Meyerhof glycolysis and pentose phosphate pathway. For a number of other bacteria, it has been shown that metabolic proteins are nonessential for growth *in vitro* and yet play vital roles *in vivo* (8, 56–58). Many of the key nutrients required for survival are provided in media used to grow these pathogens. Thus, any detrimental effect that a mutation may have may not be as easily identified *in vitro*. In contrast, *in vivo* infections provide physiological conditions for growth which are not provided via media, enabling the effects of these mutations to be detected. While the specific role of *ct148* in infectivity remains unknown, the *in vivo* data provide support for the idea that components that are seemingly nonessential for *Chlamydia* growth in tissue culture may, in fact, play significant roles in mammalian models of infection. A similar discrepancy between *in vitro* and *in vivo* growth outcomes was also observed with the *ct868*::Tn clone which disrupts *cdu1*. Cdu1 is a deubiquitinase, and it has been shown that disruption of this protein results in an increase of ubiquitination of Mcl-1 (46). Cdu1 has also been demonstrated to be capable of inhibiting host NF-κB, which represents a central regulator of numerous immune responses, including cytokine secretion and T-cell proliferation, and a vital host defense against intracellular pathogens (59–61). Overall, these observations highlight the challenges and potential discrepancies of functional studies performed in tissue culture, strengthening the necessity for analysis using *in vivo* models of infection.

The most striking phenotype was that of the observable growth and morphological deficiencies of strain *ct696*::Tn (see Fig. S2 in the supplemental material). First classified as a slow grower, approximately 8 days of growth of *ct696*::Tn were required for for detection of visible inclusions by phase microscopy, and subsequent analysis by confocal microscopy identified decreased numbers of RBs/EBs as well as the absence of the nuclear localization typically observed with *Chlamydia* inclusions. Due to its genetic location near the genes encoding the known type III secretion (T3S) effector proteins CT694 and CT695, it has been hypothesized that *ct696* may also play a role in T3S; however, studies have suggested that this may not be the case. Secretion of CT696 was not observed in previous studies employing a heterologous T3S system with Yersinia enterocolitica (62, 63), and *ct696* transcription was found to occur independently of *ct694* (64, 65). The *ct696* gene (*ctl0065*) was also disrupted using allelic exchange (33); however, growth phenotypes were not provided. CT696 is a conserved Chlamydiaceae hypothetical protein with no sequence or conserved domains shared outside the members of this family. Using the bacterial localization tool PSORTb (66), CT696 was predicted to be a cytosolic protein. While additional in-depth analysis is required to evaluate the role and function of CT696 in growth and inclusion formation, this observation provides support for these efforts as well as a good example of the utility of this transposon approach for discovery of genes important to the biology of *Chlamydia*.

Along with gaps in understanding the mechanisms of development and *in vivo* infectivity of *Chlamydia*, there is also limited information regarding the components and mechanisms for lateral gene transfer. The absence of lateral gene transfer in organisms that contain the *ct339*::Tn insertion provides compelling support for the essential contribution of this gene product to this fundamental evolutionary process. CT339 is predicted to be a multipass transmembrane protein with domain similarity to ComEC, the competence uptake protein seen in many bacteria. According to the protein family database (pfam) architecture, genes encoding proteins with this competence motif often exist near genes encoding proteins that include lactamase-superfamily domains, which are predicted to regulate the competence operon (67). Baker et al. recently showed the ComEC from *Bacillus* contains a β-lactamase-like domain within its C-terminal loop (approximately residues 494 to 763), potentially functioning as a nuclease (68). While it is widely accepted that double-stranded DNA (dsDNA) must first be converted into ssDNA for uptake and incorporation into recipient genomes, the nuclease responsible for this activity in *Bacillus* remains unknown; thus, it has been hypothesized that this lactamase-like domain within ComEC may be fulfilling this role. As a potential homolog of ComEC, a Tn disruption in the C terminus of CT339 may prevent the degradation of dsDNA into ssDNA and would decrease the ability of *Chlamydia* to undergo lateral gene transfer. However, Pfam and SMART (69) analyses of CT339 did not identify a similar lactamase-like domain near the C terminus. Rather, genome analysis of C. trachomatis identified RNase Z (CT346), a lactamase-superfamily protein neighboring CT339—suggesting that this protein may instead be fulfilling that role in the chlamydial competence system. Future studies assessing the dimerization and pore formation of CT339 and potential nuclease activity of CT346 will assist in identifying how CT339 enables DNA uptake.

Other potential components involved in DNA uptake in *Chlamydia* are less evident. Pili or pseudopili predominantly facilitate binding and transport of DNA to the bacterial surface, although no proteins sharing any sequence similarity to pilus homologs were identified in *Chlamydia*. Interestingly, Helicobacter pylori is unique among naturally transformable bacteria in that it does not use pili but instead relies on a dedicated type IV secretion system for DNA uptake (70). Gram-negative bacteria typically transport dsDNA through an outer membrane secretin channel (e.g., PilQ) and then process the dsDNA into ssDNA with a periplasmic protein, ComEA. Single-stranded DNA is then transported through inner membrane protein ComEC and into the bacterial cytoplasm, where it is bound by RecA or DprA (71). Direct pairwise BLAST analysis of C. trachomatis with numerous Gram-negative secretin homologs (PilQ) revealed sequence similarity (E values <10^−9^) to only type II and type III secretion outer membrane proteins, GspD (CT572) and YscC (CT674), respectively. Similarly, no protein with similarity to various ComEA or DprA homologs was identified through sequence-based pairwise analysis. Clearly, there is much to be learned about the *Chlamydia* DNA uptake system, although it is safe to state that, similarly to H. pylori, *Chlamydia* is likely an exception to the general DNA uptake model.

In summary, the development and characterization of a transposon system that is functional in *Chlamydia* represent a substantial advance and a valued addition to the growing repertoire of genetic manipulations in this field. Such a transposon system is expected to have an enabling effect on the discovery of biological and pathogenesis-related genes, allowing the association of single-gene insertions with evaluated phenotypes. Only a subset of the initial Tn insertion mutant clones was evaluated for *in vitro* and *in vivo* growth and infectivity. An expanded analysis of the remainder of the Tn insertional clones by the use of these and other phenotypic screens will elucidate the contribution and role of various gene products. This report has also highlighted the current inefficiency in transformation in *Chlamydia*, which will need to be overcome for large-scale mutagenesis studies. Despite this shortcoming, there is much promise for this technique and its associated applications, as evident from the support for CT339 in the fundamental evolutionary process of lateral gene transfer in *Chlamydia*. There are also high expectations that this system will be applicable and functional for studies in other *Chlamydia* species and serovars (e.g., C. muridarum and C. trachomatis serovar D), thus enabling animal studies and analysis of more clinically relevant strains for the discovery of virulence factors and potential therapeutic targets.

## MATERIALS AND METHODS

### Chlamydial strains and propagation.

C. trachomatis serovar L2 434/Bu was propagated in L929 mouse fibroblast cells (ATCC CCL-1) using RPMI 1640 medium (Invitrogen, Grand Island, NY) supplemented with 5% heat-inactivated fetal bovine serum (FBS) plus 10 μg/ml gentamicin (Fisher Scientific, Pittsburgh, PA). Briefly, L929 monolayers were grown to confluence in spinner flasks (∼8 × 10^5^ cells/ml) and infected with purified EBs at a multiplicity of infection (MOI) of 1. Cultures were allowed to grow for up to 48 h at 37°C and 5% CO_2_. Percent infectivity was assayed by IFA microscopy (described below). EBs were harvested as previously described (72) using a series of centrifugation and sonication steps to disrupt the host cells and release EBs. Once isolated, EBs were either frozen immediately or further purified using Omnipaque (Barrington, IL) and ultracentrifugation. All EBs were stored in sucrose-phosphate-glutamic acid (SPG) media at −80°C. Clonal isolates were obtained as previously described (73).

### Development of pCMA transformation plasmid.

pUC19 (GenBank accession no. L09137) was used as the initial backbone for pCMA generation and primers used are listed in Table S1 in the supplemental material. The C9 hyperactive transposase was amplified from pBADC9 (kind gift from D. Lampe of Duquesne University [3]) and cloned between the EcoRI and AatII sites. An EagI site was incorporated for subsequent promoter cloning. Initially, a vector (pCMT) that contained a tetracycline-encoding transposon was generated for application in C. muridarum by the use of pACYC184 (GenBank accession no. X06403) as the template. Primers incorporated *Himar* inverted repeats and restriction sites for cloning into the XmaI site. To remove the β-lactamase gene carried on pUC19, the transposase, tetracycline transposon, and *ori* region were amplified with primers containing NcoI sites for self-ligation. Ligase-independent cloning and HindIII sites were used to replace the tetracycline transposon with one encoding the β-lactamase and the associated promoter from pSW2 (27). To assist with selective expression of the transposase within *Chlamydia* and with less expression during plasmid propagation in E. coli, the chlamydial *ct559* promoter and ribosomal binding sites were cloned upstream of the transposase gene using the EagI site (38). The complete DNA sequence for pCMA is provided in the supplemental material. The graphical depiction of the plasmid (see Fig. S1 in the supplemental material) was generated using Savvy Scalable vector graphics (74).

10.1128/mBio.01343-19.6 TABLE S1 Primers used in this study. Download Table S1, PDF file, 0.2 MB. Copyright © 2019 LaBrie et al. 2019 LaBrie et al. This content is distributed under the terms of the Creative Commons Attribution 4.0 International license.

### Transposon mutagenesis.

The method used for transformation of C. trachomatis was modified from previously described methods (27, 39). L929 cells were seeded to confluence in 6-, 12-, or 24-well plates and allowed to adhere overnight. The *Chlamydia*-DNA transformation reaction mixture (200 μl total volume) was prepared with 10 μl of C. trachomatis L2 434/Bu EB seed stock, 1× SPG (∼1 × 10^7^ IFU), 15 μg of pCMA plasmid DNA, and 100 μl of 2× CaCl_2_ buffer (20 mM Tris-HCl [pH 7.5], 100 mM CaCl_2_), with double-distilled water (ddH_2_O) added to reach the final volume. Following gentle pipette mixing, the reaction mixtures were incubated for 30 min at room temperature (RT). The mixture was added to 12 ml of 1× CaCl_2_ buffer (10 mM Tris-HCl [pH 7.5], 50 mM CaCl_2_) before being overlaid onto a L929 monolayer that was washed once with 1× CaCl_2_ buffer (10 mM Tris-HCl [pH 7.5], 50 mM CaCl_2_). Plates were spun at 550 × *g* for 30 min at RT. Fresh RPMI medium supplemented with FBS, gentamicin, and 1 μg/ml cycloheximide was added, and the plates were incubated at 37°C and 5% CO_2_ overnight. Ampicillin (Fisher Scientific, Pittsburgh, PA) (1 μg/ml) was then added for antibiotic selection at 16 to 18 h postinfection. Cultures were kept under conditions of selection using increasing concentrations of antibiotics and serially passaged every 48 hpi. After the final passage, EBs were harvested for identification of insertion site and subsequent analyses. Transformation efficiencies were estimated using 1 × 10^7^ IFU input organism and 15 μg plasmid DNA, resulting in an average of 4 unique clonal isolates from each transformation (4 IFU/15 μg DNA). Twenty-three transformations were performed, resulting in 105 ampicillin-resistant cultures. The levels of transformation efficiency were separately assessed with five independent transformations using pGFP::SW2 plasmid under similar conditions and the same stocks of C. trachomatis as were used in transposon mutagenesis.

### Whole-genome sequencing.

DNA was extracted from purified EBs using a Qiagen blood and tissue kit (Qiagen, Valencia, CA). Manufacturer’s instructions were used with minor modifications. In brief, purified EBs were aliquoted with buffer AL before proteinase K was added and the reaction mixtures were incubated at 56°C for 1 h. After incubation, buffer AL and ethanol (96% to 100%) were added and mixed thoroughly by the use of a vortexing mixer. The reaction mixtures were added onto a provided DNeasy Mini spin column and collection tube and were centrifuged at 6,000 × *g*. Two wash steps were then performed using buffer AW1 and AW2, each with centrifugation. Finally, the reaction mixtures were incubated with buffer AE for 20 min at room temperature and centrifuged and the eluate was saved. For quality control, each sample was verified using a spectrophotometer (Denovix Ds-11 FX+) to quantify DNA. Extracted DNA was prepared for sequencing at the Genome Sequencing Core at the University of Kansas, where library preparation and further quality control were completed. Samples were multiplexed and run on an Illumina Miseq PE100 platform. Paired-end reads were generated with a Phred score (>Q30) of 95.44%. Reads were then demultiplexed and analyzed using the Geneious software suite. Total read coverage was calculated as a function of the proportion of reads that mapped to the WT reference genome (the total of chlamydial reads) over the total number of reads generated for the sample, including host DNA and other contaminant DNA. A threshold of 10 reads was created to check for the depth of coverage at each base pair (75).

### Assembly and analysis of the transposon mutant genomes.

The C. trachomatis LGV 434/Bu (“Hefty”) parental clone sequence (NCBI sequence accession no. CP019386.1) was generated through assembly guided by reference to the previously published genome Chlamydia trachomatis 434/Bu (NCBI sequence accession no. NC_010287.1). The Hefty parental genome sequence contained three mutations relative to the reference strain, namely, two nonsynonymous mutations (G946945A [H1121N] and C942546A [R274H]) and one intergenic indel (indel in lowercase; 127353/4 [AGGCCCattctaaaggccCCCTT]). After the parental draft was generated, each of the other samples were generated using the parental sequence as the reference sequence. In each case, read depth and potential loss of read continuity were considered for potential duplications or rearrangements. The reads were also mapped to the transposon sequence for each sample to ensure that the transposon was inserted into the correct site at the genome. Alignment files were generated for alignments between each mutant genome draft and the parental draft. Any mutations corresponding to these that might have accumulated through the study were annotated and verified using the reads. Host DNA contamination within each sample was mitigated using the reference-guided assembly techniques. Transposon insertion sites were analyzed both through the use of the reference-guided mapping of the genomes and separately through the use of reference-guided assembly of the raw reads to the transposon sequence. In each case, these were analyzed separately from the hypothesized insertion sites to prevent bias. The read quality of the transposon assemblies was proportional to the size of each genome in every case. Plasmid sequences were generated for each sample by using the C. trachomatis LGV plasmid (NCBI sequence accession no. NC_021051.1). Plasmid gene assignments incorporated both *pgp1-8* (42) and *cds1-8* (76) nomenclature. All alignments were performed in Geneious using global alignment with free end gaps and a cost matrix corresponding to 65% similarity. Completed sequences were submitted to GenBank and assigned accession numbers (see “Data accessibility” section below). The transposon mutant genomes were evaluated for new single nucleotide polymorphisms (SNPs), and no mutations were discovered after the transformation and cultivation processes. While all studies were performed using C. trachomatis LGV 434/Bu, C. trachomatis serovar D gene nomenclature is utilized due to recognition and field familiarity.

### Assembly and analysis of the recombinant progeny genomes.

Each recombinant genome was generated using assembly guided by reference to the OmpA parental genome. Genomes were then aligned to both parental genomes and scanned for regions of homology using the Geneious global alignment tool with free end gaps and a cost matrix corresponding to 65% similarity. Initial sequence assembly was verified through assembly guided by reference to the minority parent. Regions of homology were annotated, and margins were estimated based on similarity to the individual parents.

### *In vitro* growth analysis of Tn mutants.

For temporal assessment of progeny production, L929 mouse fibroblast cells were infected in triplicate with C. trachomatis parental or Tn mutant clones with a multiplicity of infection (MOI) of 0.5 in 24-well plates. At 18, 24, 30, 36, and 42 h postinfection (hpi), wells were washed once with Hanks’ balanced salt solution (HBSS) and then immediately frozen in 1× SPG at −80°C within the 24-well plate. The 24-well plates were then thawed on ice and transferred to 1.5-ml Eppendorf tubes and sonicated in a Q500 Qsonica cuphorn sonicator (Qsonica) with the following settings: 75% amplitude; 4 pulses; 30-s ON/30-s OFF cycle in chilled (∼5°C) water. To determine titers of inclusion-forming units, samples were then serially diluted and used to infect confluent monolayers of L929 mouse fibroblast cells in 96-well μClear CellStar plates (catalog no. 655090; Greiner Bio-One, Frickenhausen, Germany) or 8-well μ-Slides (Ibidi, Martinsried, Germany). After 24 h postinfection, the cells were washed with HBSS and then fixed with 100% methanol. Cells were washed with phenol-free HBSS before staining was performed with a MicroTrak C. trachomatis culture confirmation kit (Trinity Biotech, Wicklow, Ireland). After at least 1 h of room temperature incubation, 1 μM DAPI (4′,6-diamidino-2-phenylindole) diluted in phosphate-buffered saline (PBS) was added and allowed to stain wells for 10 min. A final overlay of 0.1 M Tris-glycerol was added, and cells were imaged on an Olympus IX71 inverted microscope (Olympus, Waltham, MA) with both 10× and 40× objectives. Inclusions were enumerated manually for each sample by immunofluorescence microscopy. For calculation of progeny production, titers were also determined for the inoculum that was initially used to infect the 24-well plates at the time of infection to incorporate subtle differences in starting infectivity for each mutant relative to the parental strain (see Fig. S4 in the supplemental material). To normalize variations in Tn mutant progeny production that would result from differences in starting infectivity levels, the ratio of starting inoculum of the parental strain to the starting inoculum of the Tn mutant was individually multiplied by each Tn mutant’s titers at each time point (18, 24, 30, 36, or 42 hpi). Data are shown as means and standard deviations (SD) for triplicate wells, generated in GraphPad Prism 7, and statistical differences were calculated using the two-tailed Student's *t* test. With a *P* value of <0.05, *ct732*::Tn had significantly reduced progeny production at 24, 30, 36, and 42 hpi; *ct590*::TN and *ct036*::Tn had significantly reduced progeny production at 24, 30, and 42 hpi; *ct350*::Tn had significantly reduced progeny production at 30 and 42 hpi; *ct153*::Tn and *ct550*::Tn had increased progeny production at 24 hpi; *ct339*::Tn had increased progeny production at 24 and 42 hpi; and *ct148*::Tn had increased progeny production at 30 and 42 hpi.

### Confocal immunofluorescence microscopy.

L929 cells were grown to confluence in an 8-well ibiTreat μ-Slide (Ibidi, Martinsried, Germany) and were infected with the respective C. trachomatis/Tn mutants. At ∼24 hpi, infected cells were fixed with 100% methanol for 10 min at RT. Cells were washed once with HBSS and again with PBS and then stained using the preparation supplied in the MicroTrak C. trachomatis culture confirmation test (Syva Co., Palo Alto, CA) (180 μl) diluted 1:40 in PBS for 1 h 50 min at RT in the dark or overnight at 4°C. A 2-μl volume of 1 μM DAPI (4′,6-diamidino-2-phenylindole) diluted 1:100 in PBS was then added to wells and allowed to stain for 10 min at RT in the dark. The stain was then removed, and the cells were washed with PBS. A final overlay of Vectashield antifade mounting medium (Burlingame, CA) was added, and slides were stored at 4°C in the dark until the imaging step. Cells were visualized on an Olympus IX81/3I spinning disk confocal inverted microscope at ×150 magnification and captured on an Andor Zyla 4.2 scientific complementary metal oxide semiconductor (sCMOS) camera (Belfast, Northern Ireland). Microscope and camera were operated using SlideBook 6 software (Intelligent Imaging Innovations, Denver, USA). Exposure time remained consistent for all fields captured, with the exposure time for DAPI set at 2 s, for OmpA at 4 s, and for cytoplasm at 6 s. Three to seven Z stack images were taken at 0.3-μm intervals per cell imaged. Images were processed in SlideBook 6, and No Neighbors deconvolution (with a subtraction constant of 0.4) was applied to all images.

### *In vitro* inclusion size analysis for Tn mutants.

L929 mouse fibroblast cells were infected at low MOI (∼10% of cells infected) in triplicate on two separate 96-well plates with parental population or a clonal population of Tn mutant Chlamydia trachomatis. At 24 hpi, cells were fixed with methanol for 10 min. Cells were then stained using a MicroTrak C. trachomatis culture confirmation kit for 1 h at room temperature. DAPI (4′,6-diamidino-2-phenylindole) (1 μM) was diluted in PBS and allowed to stain wells for 10 min. A final overlay of 0.1 M Tris-glycerol was added, and cells were imaged on an EVOS FL Auto 2 microscope (Thermo Scientific, Waltham, MA) using a 20× objective. A total of 16 fields were imaged for 6 wells for each sample, including more than 200 inclusions per sample (range, approximately 200 to 1,000). Each inclusion area was then measured using MIPAR (MIPAR, Worthington, OH). Within MIPAR, segmentation of inclusions was performed by applying a threshold to images to identify inclusions based on a range of pixel values that was standardized to parental images. After segmentation of inclusions, the total area was measured and quantified as the total number of pixels. This output was then exported to GraphPad, and statistical analysis was performed. Statistical analysis consisted of a one-way analysis of variance (ANOVA) with Dunnett’s multiple-comparison test (statistical significance *P* value, <0.05).

### Mouse transcervical infections.

Female C57BL/6 mice (6 to 8 weeks old) were purchased from Jackson Laboratories and housed in accordance with the requirements specified by the University of Kansas Institutional Care and Use Committee. Mice were treated subcutaneously with 2.5 mg medroxyprogesterone acetate (Depo-Provera, Pfizer, NY) upon arrival (day −7). Fresh aliquots of parental or Tn mutant C. trachomatis clones were diluted in sucrose-phosphate-glutamic acid (SPG) buffer and kept on ice until use. Using a nonsurgical embryo transfer device (NSET; ParaTechs, Lexington, KY), mice were inoculated transcervically with 10 μl diluted stock (final concentration, 5 × 10^5^ IFU/mouse) by insertion of the device into the genital tract beyond the opening of the cervix. For each infection, samples used to infect mice were also evaluated for titer to ensure delivery of the expected infectious dose. The infections and analyses performed with *ct868*::Tn were repeated from the initially reported experiments (46).

### ddPCR assessment of *Chlamydia* infection *in vivo*.

Mice were humanely euthanized 5 days postinfection, and the genital tracts were collected in SPG. Uterine horns were homogenized using a rotor/stator homogenizer (Biospec, Bartlesville, OK). Aliquots (100 μl) were frozen at −20°C until use. The remaining stocks of homogenized tissues were stored at −80°C. DNA isolation was performed using the 100-μl aliquots and a DNeasy blood and tissue kit (Qiagen). Isolated DNA was then used for droplet digital PCR (ddPCR) analyses. Primers and probes for C. trachomatis *secY* and murine *rpp30* (see Table S1 in the supplemental material) were used with ddPCR Supermix for Probes (Bio-Rad, Hercules, CA) to set up the PCRs. Oil-for-Probes droplet emersions were generated using a droplet generator cassette (Bio-Rad). The PCR conditions were as follows: 95°C for 10 min and 40 cycles of 94°C for 30 s and 98°C for 10 min followed by cooling to 4°C. Fluorescent reads of individual droplets were calculated after PCR was performed using a QX200 droplet reader (Bio-Rad). Data were analyzed using QuantaSoft Software (Bio-Rad), and the results are reported as log_10_ ratios of *Chlamydia* DNA to host DNA (*secY*/*rpp30* copies/μl). Box and whisker scatter plots were generated in GraphPad Prism 7, and recoverable organisms were compared using unpaired multiple *t* tests with no correction for multiple comparisons.

### Bioinformatic analysis of CT339 and chlamydial competence.

BLAST analyses and subsequent queries within the Conserved Domain Database resulted in hits within the competence superfamily and multidomain hits for ComEC. Multiple-sequence alignments were generated using ClustalW and 10 variable species hits within the Clusters of Orthologous Groups of proteins (COG0658) (77). The following proteins were used (NCBI accession numbers shown in parentheses): competence protein ComEC family protein Shigella dysenteriae 1617 (YP008850592.1), competence locus E Helicobacter pylori Hp P-26 (EJC51989.1); competence protein ComEC Enterococcus faecium DO (YP006375852.1); ComE *Synechocystis* sp. PCC 6803 (BAA17126.1); ComEC Listeria monocytogenes EGD-e (CAC99560.1); ComE operon protein 3 Bacillus subtilis subsp. *subtilis* strain 168 (NP 390435.1); hypothetical protein AGR C 2573 Agrobacterium tumefaciens sp. strain C58 (NP354400.1); competence protein Neisseria meningitidis MC58 (NP273744.1); hypothetical protein CT339 Chlamydia trachomatis D/UW-3/CX (NP 219846.1); ComEC/Rec2 family protein Clostridium botulinum A strain ATCC 3502 (YP 001255462.1); ComEA Neisseria meningitidis (CAB44958); ComEA protein Helicobacter bilis (WP_004084273); competence ComEA Shigella dysenteriae 1617 (EFP70107); competence protein ComGA Enterococcus faecium (WP_002304768); competence protein ComGA Listeria monocytogenes (WP_009933475); ComGA Bacillus subtilis (BAA12533); (multispecies) competence protein ComGB (WP_002286180); (multispecies) competence protein ComGB *Bacilli* (WP_048681721); competence protein ComGB Listeria monocytogenes (WP_010990101). (multispecies) type IV pilus secretin PilQ *Neisseria* (WP_016686778); type 4 pilus secretin PilQ (outer membrane porin) Shigella dysenteriae 1617 (AHA67697); (multispecies) pilus assembly protein PilE *Neisseria* (WP_002214937); and ComGC Bacillus subtilis (BAA12535). The following five hydropathy prediction programs were used to predict topology for CT339: HMMTOP v2.0 (78); MEMSAT-SVM (79); TMHMM2.0 (80); TOPPRED (81); and SPLIT (82). Transmembrane illustrations were generated and modified using Protter v1.0 (83).

### Generation of recombinant clones for assessment of bacterial competence.

All experiments performed with tetracycline-resistant *Chlamydia* as described in this work were reviewed and approved by the National Institutes of Health Recombinant DNA Advisory Committee (University of Washington). Recombination experiments were performed as previously described (47). Briefly, sets of individual shell vials were seeded with McCoy cells and subsequently coinfected with the following combinations of drug-resistant strains: C. trachomatis L1/*tet*^r^ (NCBI accession number ACUI01000000), C. trachomatis serovar F/*tet*^r^, and either C. trachomatis LGV2 (*ct383*/*4*::Tn *bla*) or C. trachomatis LGV2 *ct339*::Tn *bla*. Cultures were incubated for 40 h postinfection in the absence of antibiotics and were then detached using −80°C/37°C freeze-thaw. Recombinants were isolated by infection of 96 new shell vial monolayers with 1 ml freeze-thaw lysates and treatment with both penicillin and tetracycline and were passaged until dually resistant clones were detected. Recombinant clones were propagated and cloned by limiting dilution and subsequently evaluated for identification of serovar*-*specific OmpA. DNA from clones was harvested and subjected to WGS as described above.

### Data accessibility.

Completed sequences were submitted to GenBank and assigned the following accession numbers (shown in parentheses): *CT015*::Tn (NHAT01000000); *CT036*::Tn (NHAU01000000); *CT153*::Tn (NHAV01000000); *CT333*::Tn (NHAW01000000); *CT339*::Tn (NHAX01000000); *CT392*::Tn (NHAY01000000); *CT550*::Tn (NHAZ01000000); *CT819*::Tn (NHBA01000000). The pCMA vector was completely sequenced and deposited (GenBank accession no. MN177722).
