# Supplementary material for: Transposon Mutagenesis in Chlamydia trachomatis Identifies CT339 as a ComEC Homolog Important for DNA Uptake and Lateral Gene Transfer
Source: mBio. 2019 Aug 6;10(4):e01343-19. doi: 10.1128/mBio.01343-19 (PMC6686042; doi:10.1128/mBio.01343-19)
Supplement: FIG S1 [file mBio.01343-19-sf001.pdf]

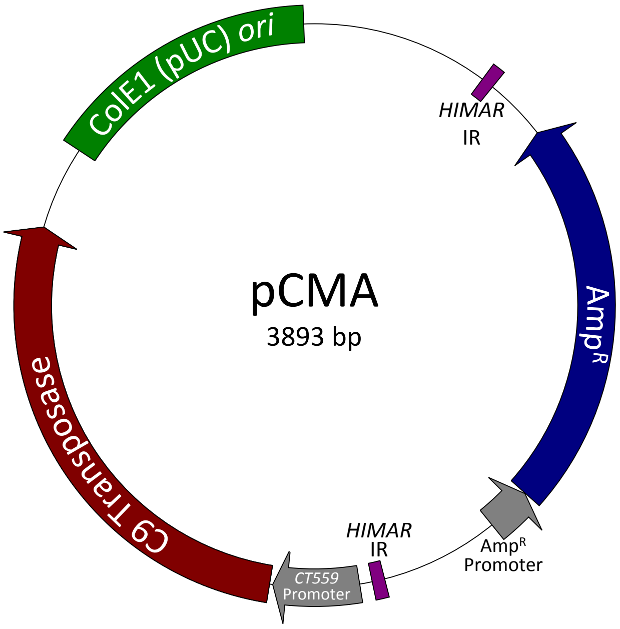

Supplementary Figure 1. Plasmid map of pCMA. The C9 hyperactive variant of the *Himar1* transposon was cloned downstream of the *Chlamydia ct559* promoter. β-lactamase (*bla*) and associated promoter was cloned from the chlamydial shuttle vector pGFP::SW2 (29). This antibiotic marker is flanked by *Himar1* inverted repeats to generate the transposon.
