# Supplementary material for: Transposon Mutagenesis in Chlamydia trachomatis Identifies CT339 as a ComEC Homolog Important for DNA Uptake and Lateral Gene Transfer
Source: mBio. 2019 Aug 6;10(4):e01343-19. doi: 10.1128/mBio.01343-19 (PMC6686042; doi:10.1128/mBio.01343-19)
Supplement: FIG S2 [file mBio.01343-19-sf002.pdf]

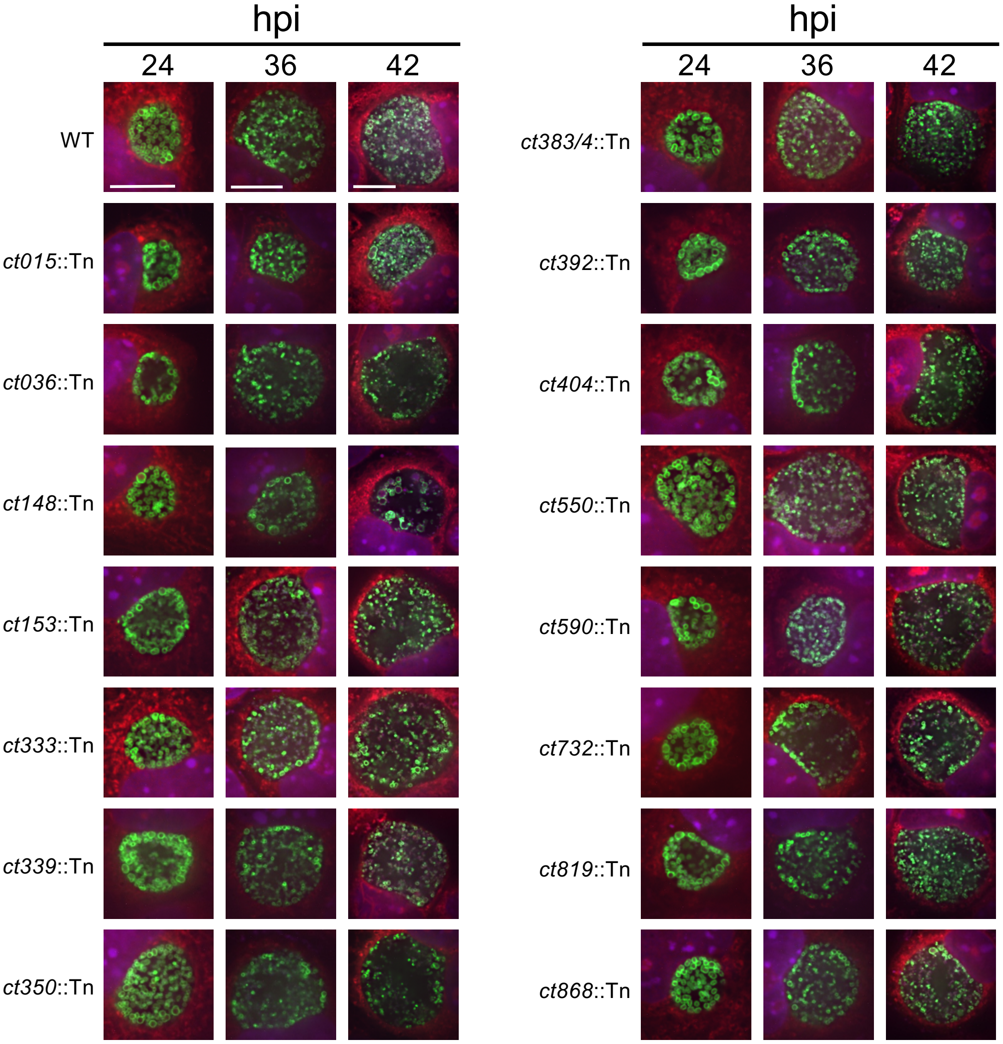

Supplementary Figure 2. Confocal microscopy of Tn mutants at 24, 36, and 42hpi. L929 cells were infected with WT and mutant *C.* trachomatis at 24, 36, or 42 hpi. Samples were stained with DAPI (blue), and a *C. trachomatis* Culture Confirmation Test for MOMP (green), and host cytosol (red). Images were captured at 150X magnification with single z-plane displayed. Scale bar = 10μm and is consistent within time points.
