# Supplementary material for: Transposon Mutagenesis in Chlamydia trachomatis Identifies CT339 as a ComEC Homolog Important for DNA Uptake and Lateral Gene Transfer
Source: mBio. 2019 Aug 6;10(4):e01343-19. doi: 10.1128/mBio.01343-19 (PMC6686042; doi:10.1128/mBio.01343-19)
Supplement: FIG S4 [file mBio.01343-19-sf004.pdf]

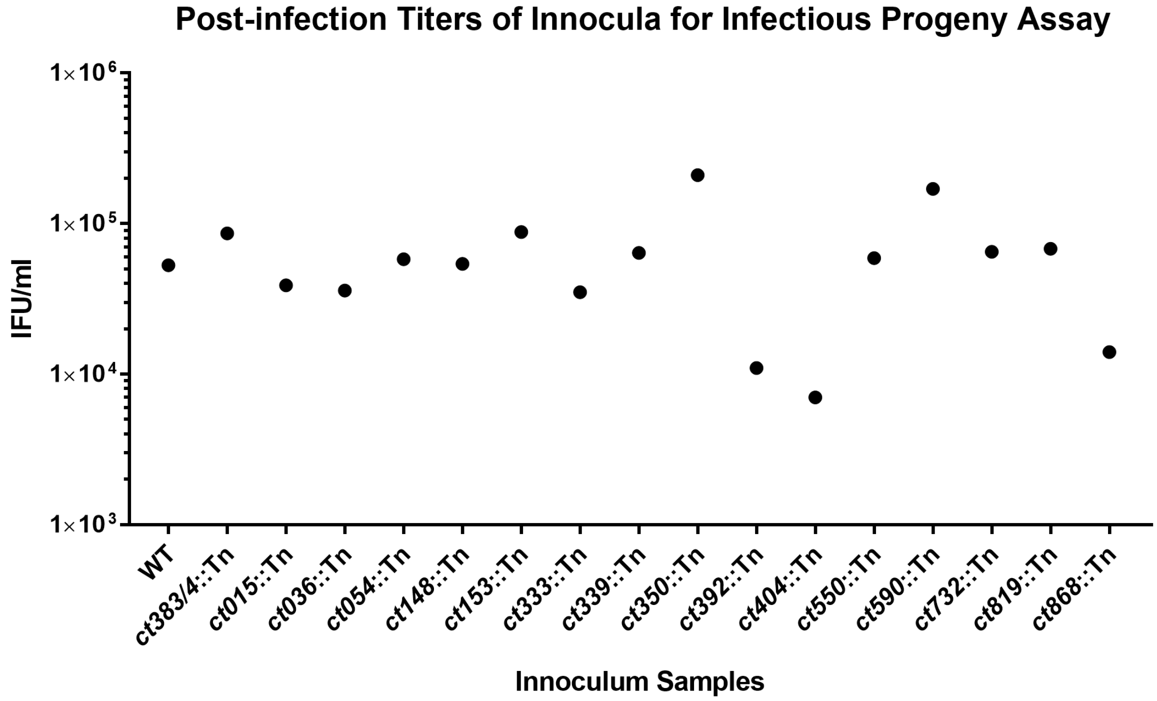

Supplementary Figure 4. Post-infection titers of innocula for infectious progeny assay. The innocula used for the progeny production assay was titered to reflect the specific dose used to infect the 24-well plates. L929 cells were infected with innocula in a 96-well plate and incubated at 37C and 5% CO2. At 24 hours post-infection, cells were washed, fixed with methanol, washed, and stained with DAPI (nucleic acid) and a *Chlamydia trachomatis* Culture Confirmation Test to view inclusions (anti-MOMP) and to stain the host cytosol. Samples were titered to determine inclusion forming units per ml (IFU/ml).
