# Supplementary material for: Transposon Mutagenesis in Chlamydia trachomatis Identifies CT339 as a ComEC Homolog Important for DNA Uptake and Lateral Gene Transfer
Source: mBio. 2019 Aug 6;10(4):e01343-19. doi: 10.1128/mBio.01343-19 (PMC6686042; doi:10.1128/mBio.01343-19)
Supplement: FIG S3 [file mBio.01343-19-sf003.pdf]

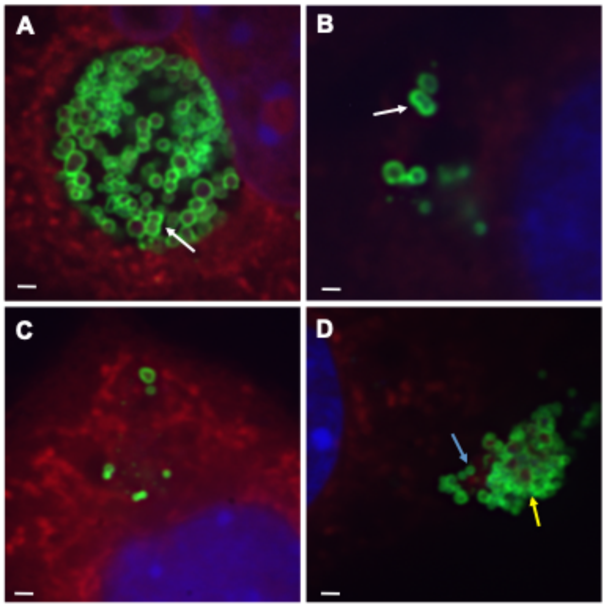

Supplementary Figure 3. Confocal microscopy of *ct696*::Tn mutant. L929 mouse fibroblasts were infected with either *C. trachomatis* parental or *ct696*::Tn mutant. Infections were fixed at 24 hpi and immunofluorescently stained. Images were acquired at 150X magnification with deconvolution for enhanced image resolution. **(A)** WT *C. trachomatis* inclusion showing normal RB division (white arrow). ***(*B)** *ct696*::Tn showing normal RB division (white arrow). **(C)** Dispersed localization of *ct696*::Tn within the cytosol. **(D)** Aggregating form of *ct696*::Tn. Coccoid shaped RBs (blue arrow) are observed external to the aggregation (yellow arrow). Blue: DAPI, nucleus; Red: Evan's Blue, cytoplasm; Green: OmpA, *C. trachomatis* organisms. Scale bar = 1μm.
