# Supplementary material for: Transposon Mutagenesis in Chlamydia trachomatis Identifies CT339 as a ComEC Homolog Important for DNA Uptake and Lateral Gene Transfer
Source: mBio. 2019 Aug 6;10(4):e01343-19. doi: 10.1128/mBio.01343-19 (PMC6686042; doi:10.1128/mBio.01343-19)
Supplement: FIG S5 [file mBio.01343-19-sf005.pdf]

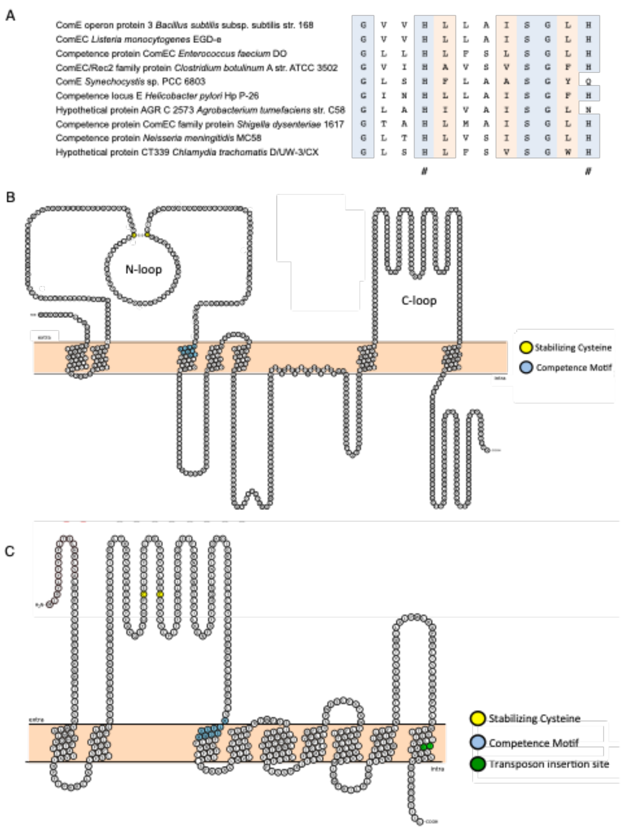

Supplementary Figure 5. CT339 has a predicted metal binding motif and similar topology to ComEC. **(A)** Multiple sequence alignment of 10 competence proteins identified as containing the multi-domain ComEC from the Conserved Domain Database. COG0658 was used to identify 10 bacterial species with similar competence domains with an E-value < 6e-3. Hashtags indicate conserved histidine metal-binding motifs. Conserved hydrophobic residues are highlighted in orange. Hydropathy plots of **(B)** ComEC from *Bacillus subtilis* and **(C)** CT339 from *Chlamydia*. Both predicted competence proteins display a large extracellular N-terminal loop stabilized by two cysteines (highlighted in yellow) and an extended C-terminal loop. The competence domain as identified by the conserved domain database is highlighted in blue and the transposon insertion site is highlighted in green.
