# Supplementary material for: Transposon Mutagenesis in Chlamydia trachomatis Identifies CT339 as a ComEC Homolog Important for DNA Uptake and Lateral Gene Transfer
Source: mBio. 2019 Aug 6;10(4):e01343-19. doi: 10.1128/mBio.01343-19 (PMC6686042; doi:10.1128/mBio.01343-19)
Supplement: TABLE S1 [file mBio.01343-19-st001.pdf]

Supplementary Table 1: Primers used in this study.

pCMA Plasmid Design (5'-3')	
**Gene**	**Primer Sequence and Designation**
C9 Transposase (**EcoRI/AatI**)	GCG**GAATTCCGGCCG**ATGGAAAAAAAGGAATTTCGTGTTTTG
	GC**GACGTC**TTATTATTCAACATAGTTCCCTTCAAG
pCMT Tet fragment with HIMAR IR (**XmaI**)	GC**CCCGGG**TAACAGGTTGGCTGATAAGTCCCCGGTCTTTGGTAGCTCAGAGAACCTTCG
	GC**CCCGGG**TAACAGGTTGGCTGATAAGTCCCCGGTCTCTGGAGATGGCGGACGCGATGG
pCMT amplicon (**NcoI**)	NNNNNN**CCATGG**ACTGTCAGACCAAGTTTACTC
	NNNNNN**CCATGG**CTGACGTCTTATTATTCAACATAG
pSW2 *β-lactamase* (LIC-**HindIII**)	GACCATGATTACGCCAAGCTAACAGGTTGGCTGATAAGTCCCCGGTCTGACGCTCAGTGG
	CAGCTTATCATCGAT**AAGCTT**AGACGTCAGGTGGCAC
CT559 promoter (LIC - **EagI**)	ACCGAGCTCGAATTC**CGGCCG**CCCATTCGTTCGTTCTAG
	TTCCTTTTTTTCCAT**CGGCCG**CACACCTTTTCACTCAC

Droplet Digital PCR (ddPCR) (5'-3')	
**Gene**	**Primer Sequence and Designation**
secY	TAAAAAGCCGTGTCATTCGTCC
	TCGGCTTCAATCATTGTACAGC
secY Probe	/56-FAM/TAATTTACG/ZEN/CTTCCCTTGATCCGGC/3IABkFQ/
rpp30	CTCTTCCAGTGTGCAAGAAAGC
	AGTGACTGATGAGCTACGAAGG
rpp30 Probe	/5HEX/TGAGACGAGTCCTGAGTCTC/3IABkFQ/
